# Anti-cancer activity of synthetic gefitinib-1,2,3-triazole derivatives against Hela cells via induction of apoptosis

**DOI:** 10.3389/fchem.2025.1456743

**Published:** 2025-01-24

**Authors:** Zhihong Hu, Xixi Hou, Yongjing Ren, Ziyuan Wu, Dong Yan, Hong Chen, Lan Wang

**Affiliations:** ^1^ College of Basic Medicine and Forensic Medicine, Henan University of Science and Technology, Luoyang, China; ^2^ The First Affiliated Hospital, College of Clinical Medicine of Henan University of Science and Technology, Luoyang, China; ^3^ Luoyang Key Laboratory of Organic Functional Molecules, College of Food and Drug, Luoyang Normal University, Luoyang, China

**Keywords:** cervical cancer, gefitinib, 1,2,3-triazole, cell cycle, apoptosis

## Abstract

Cervical cancer ranks as the fourth most common cancer among women. However, the current treatments have significant side effects and limited therapeutic effects on advanced diseases, so it is necessary to discover better treatments for cervical cancer. The current study investigated the potential anticancer effects of a series of gefitinib-1,2,3-triazole derivative on Hela cells. Among the investigated, the target compound **c13** showed good anticancer activity against Hela cells (IC_50_ = 5.66 ± 0.35 μM) compared with gefitinib (IC_50_ = 14.18 ± 3.19 μM). Moreover, compound **c13** significantly inhibited the colony formation ability of Hela cells in a dose-dependent manner, accompanied by morphological changes in HeLa cells. Further investigations demonstrated that compound **c13** triggered cell apoptosis and arrested the cell cycle at the G2/M phase in Hela cells. In addition, western blot analysis revealed that compound **c13** upregulated the Bax/Bcl-2 ratio, and increased the levels of active caspase 3 and PARP1 cleavage, which suggested the involvement of the mitochondrial pathway in compound **c13**-induced apoptosis. In brief, these results indicated that compound **c13** is a promising compound for the treatment of cervical cancer.

## 1 Introduction

According to Global Cancer Statistics 2020 years, cervical cancer (CC) is the fourth most common malignant disease and is a major threat to women’s health worldwide, with approximately 604,000 new cases and 342,000 deaths each year worldwide (Sung et al., 2021). The commonly used treatment methods for cervical cancer include surgical treatment, radiotherapy, chemotherapy, etc (Johnson et al., 2019). However, most of the above treatment methods have certain side effects, such as gastrointestinal reactions, bone marrow transplantation, and liver and kidney function damage, which to some extent reduces the quality of life of patients with cervical cancer (Ferrall et al., 2021). Therefore, we urgently need to discover effective and safer anticancer agents to provide better treatment results and reduce side effects for patients.

Gefitinib, one of the first-generation epidermal growth factor receptor (EGFR)-tyrosine kinase inhibitors, was approved by the US Food and Drug Administration (FDA) to treat metastatic non-small cell lung cancer in 2015 (Kazandjian et al., 2016). Nowadays, gefitinib has been reported to be used to treat certain types of cancer with high expression of EGFR, including cervical cancer (Sharma et al., 2013; Krishna et al., 2023). Meanwhile, one study reported that treatment of gefitinib caused Hela cells apoptosis and cell cycle arrest, and also suppressed epithelial-mesenchymal transition (EMT) via the Wnt/β-catenin signaling pathway (Zheng et al., 2019). Another study revealed that gefitinib induced anoikis to exert the anti-proliferative effect on cervical cancer cell lines including Hela cells and C33A cells (Jung et al., 2024). According to previous studies, gefitinib exerts a potent anti-cancer effect against cervical cancer.

1,2,3-triazole is a well-known scaffold among nitrogen-containing heterocycles that could interact with different biological targets by forming various non-covalent bonds such as hydrophobic interactions, hydrogen bonding, van der Waals forces, and so on (Bonandi et al., 2017; Bozorov et al., 2019). Therefore, the compounds containing 1,2,3-triazole scaffold usually have various pharmacological properties such as anti-cancer, anti-inflammation, anti-bacterial, anti-viral, and antitubercular effects (Alam, 2022; Deng et al., 2022; Feng et al., 2021). The characteristics of 1,2,3-triazole enable it to simulate various functional groups, so 1,2,3-triazole has already been widely used as a bioisostere in drug discovery (Bonandi et al., 2017). In our previous studies, gefitinib was modified by 1,2,3-triazole to improve its anticancer activity against lung cancer cells (Liu et al., 2024). In addition, erlotinib derivatives incorporating various 1,2,3-triazole moieties displayed remarkable antitumor activity than erlotinib on different kinds of cancer cell lines. Especially, compound **3d**, erlotinib modified with (3,5-dibromobenzyl)-1H-1,2,3-triazole, exhibited significant inhibitory effects against diverse cancer cell lines (Mao et al., 2022). Also, we introduced 1,2,3-triazole groups with different substituents to obtain icotinib derivatives, among which compound **a12** showed effective antitumor activity in non-small-cell lung cancers cells as a potent inhibitor for EGFR with IC_50_ value of 1.49 μM (Mao et al., 2020). Therefore, we introduced 1,2,3-triazol moiety to gefitinib to obtain a series of gefitinib-1,2,3-triazole derivative. Given the promising antitumor effect of novel gefitinib-1,2,3-triazole derivatives, the current study was designed to evaluate the cancer-combating properties of gefitinib-1,2,3-triazole derivatives on Hela human cervical cancer cell line, which aimed to find new potential anticancer drugs for cervical cancer.

## 2 Chemistry

**SCHEME 1:**
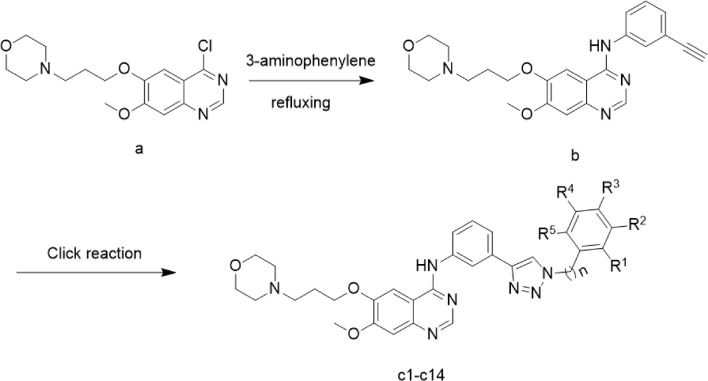
Synthesis route of Gefitinib-1,2,3-triazole derivatives.

The preparation of the target compounds is outlined in Scheme 1. Compound **c1-14** was synthesized using the click chemistry approach as previously described (Liu et al., 2024). Compound **b** was synthesized by reacting 4-(3-((4-chloro-7-methoxyquinazolin-6-yl)oxy) propyl) morpholine (1) with 3-aminophenylacetylene. The target compounds **c1-14** was then obtained through a click reaction between compound **b** and azide compounds. These reactions were conducted under mild conditions and were straightforward to perform. The structures of the key intermediates and the target compound were confirmed using nuclear magnetic resonance (^1^H NMR and ^13^C NMR) and high-resolution mass spectrometry (HR MS). The structural characterization of compounds **c1-14** were provided in supplementary information.

## 3 Results

### 3.1 Gefitinib derivatives inhibited the cell viability of Hela cells

**FIGURE 1:**
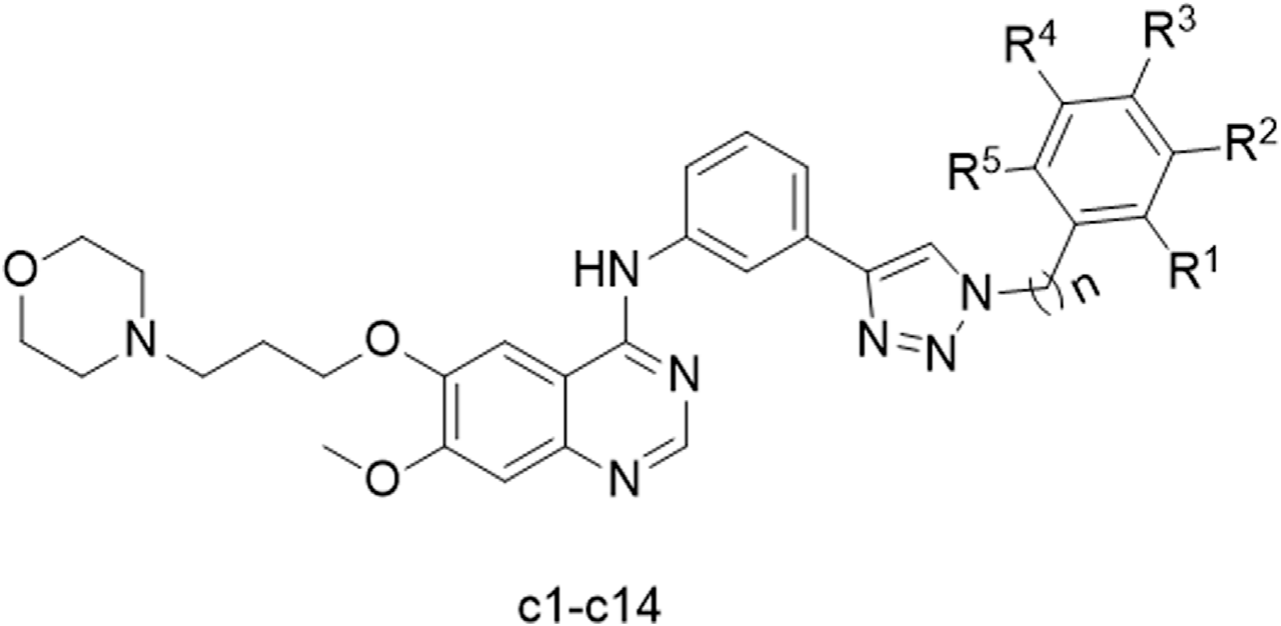
Structure of synthesized compounds **c1-14**.

To improve the anticancer effect of gefitinib on Hela cells, we introduced a 1,2,3-triazole motif into gefitinib. According to our previous research, the synthesis of 1,2,3-triazole analogs of gefitinib was successfully carried out. The structure of the synthesized compounds is shown in Figure 1.

To investigate the anti-proliferative effects of Gefitinib derivatives **c1-14** on Hela cells, the cell viability was detected using the MTT method after being treated with compounds **c1-14** for 48 h. Doxorubicin was used as positive control. Cell viability of gefitinib and its derivatives at 10 μM were valued and summarized in Table 1. As shown in Table 1, after treatment with 10 μM of gefitinib, the cell viability of Hela cells was 75.87% ± 2.88%. And compound c13, N-(3-(1-(3,5-dibromobenzyl)-1H-1,2,3-triazol-4-yl)phenyl)-7-methoxy-6-(3-morpholinopropoxy)quinazolin-4-amine), was suggested as the most highly active compound against Hela cells. Therefore, compound c13 was selected for further investigation.

**TABLE 1:** Antiproliferative activities of the synthesized compounds c1-14 against Hela cells.

Compd no.	n	R1	R2	R3	R4	R5	% Cell viability (at 10 μM)
c1	1	H	Cl	F	H	H	83.83 ± 0.90^**^
c2	1	Br	H	H	H	H	82.77 ± 2.29^***^
c3	1	H	F	H	H	H	77.17 ± 2.75^**^
c4	1	Br	H	H	F	H	73.80 ± 3.37^**^
c5	1	F	H	Br	H	H	93.13 ± 2.03^**^
c6	1	H	Cl	F	H	H	98.00 ± 1.45
c7	1	F	H	H	H	Cl	70.94 ± 6.71^*^
c8	1	NO_2_	H	H	H	H	78.37 ± 2.76^**^
c9	1	H	H	NO_2_	H	H	97.40 ± 0.65^*^
c10	0	OCH_3_	H	NO_2_	H	H	70.49 ± 1.24^***^
c11	1	H	H	Br	H	H	88.93 ± 2.89^**^
c12	1	CN	H	H	H	H	76.40 ± 1.15^**^
c13	1	H	Br	H	Br	H	32.61 ± 8.61^**^
c14	1	H	H	CH_3_	H	H	86.63 ± 1.32^**^
Gefitinib	-	-	-	-	-	-	75.87 ± 2.88^**^
Doxorubicin							11.95 ± 1.20^***^

All values are given as means ± SEM (n = 3).

**FIGURE 2:**
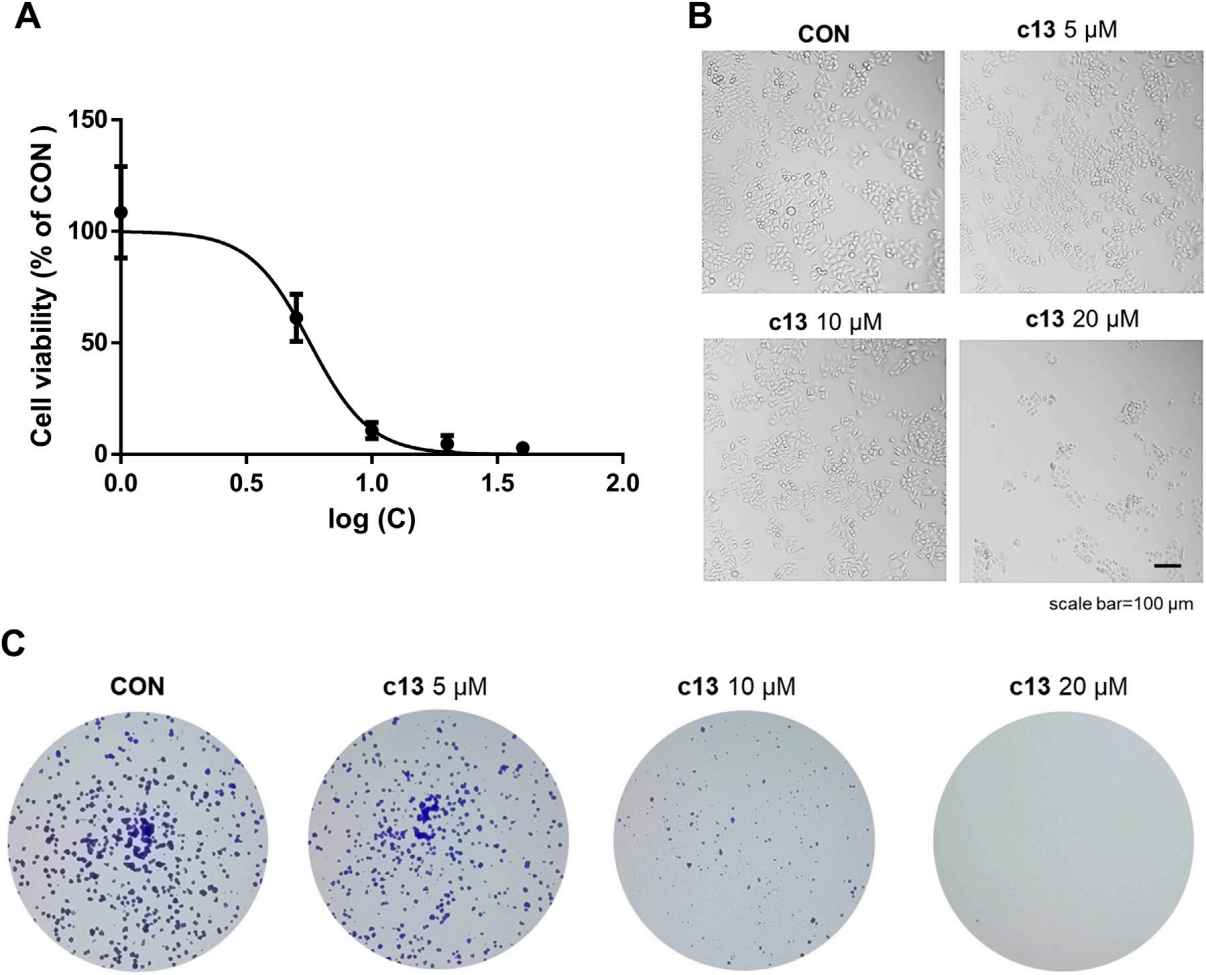
Anti-proliferative Effects of compound c13 on Hela cells. **(A)** Dose-dependent cytotoxicity of compound **c13**, assessed at concentrations ranging from 0 to 40 μM on HeLa cells. Cell viability was plotted against the logarithm of compound **c13** concentrations (n = 3). **(B)** Morphological profiles of Hela cells were taken by microscope after treatment with compound **c13** at different doses (0, 5, 10, and 20 μM) (n = 3). **(C)** Representative photomicrographs of plate colony formation assay data with compound **c13** at different doses (0, 5, 10, and 20 μM) (n = 3).

To define the effects of compound **c13** on cancer cell proliferation, the half-maximal inhibitory concentration (IC_50_) of compound **c13** in Hela cells was calculated. Hela cells were treated with compound **c13** at different concentrations (0 μM, 1 μM, 5 μM, 10 μM, 20 μM, and 40 μM) respectively for 48 h, and the cell viability was detected using the MTT method. According to the results, compound **c13** showed an IC_50_ value of 5.66 ± 0.35 μM against Hela cells (Figure 2A), while the IC_50_ of gifitinib in Hela cells was 14.18 ± 3.19 μM. We further investigated the effects of compound c13 (0, 10, 20, and 40 μM) on the viability of HEK293T cells (human embryonic kidney epithelial cells), NIH3T3 cells (mouse embryonic fibroblasts), and Hela cells. The experimental results are shown in the Supplementary Figure S1, where compound c13 significantly inhibited the proliferation of Hela cells. In contrast, its effect on the proliferation of HEK293T and NIH3T3 cells was minimal, with both IC_50_ values exceeding 100 μM. Therefore, compound c13 selectively inhibits the proliferation of Hela cervical cancer cells without significant toxic effects on normal cells, HEK293T cells and NIH3T3 cells, indicating its higher safety profile.

Also, severe morphological changes of Hela after treatment of compound **c13** were observed, including cell shrinkage and an increase in floating dead cells (Figure 2B). To validate the impact of compound **c13** on cell proliferation, plate clone formation assay was performed. As shown in Figure 2C, compound **c13** significantly inhibited the colony formation ability of Hela cells in a dose-dependent manner. Taken together, these data suggested that compound **c13** possessed an inhibitory effect on the proliferation of Hela cells.

### 3.2 Compound c13 triggered cell cycle arrest in Hela cells

**FIGURE 3:**
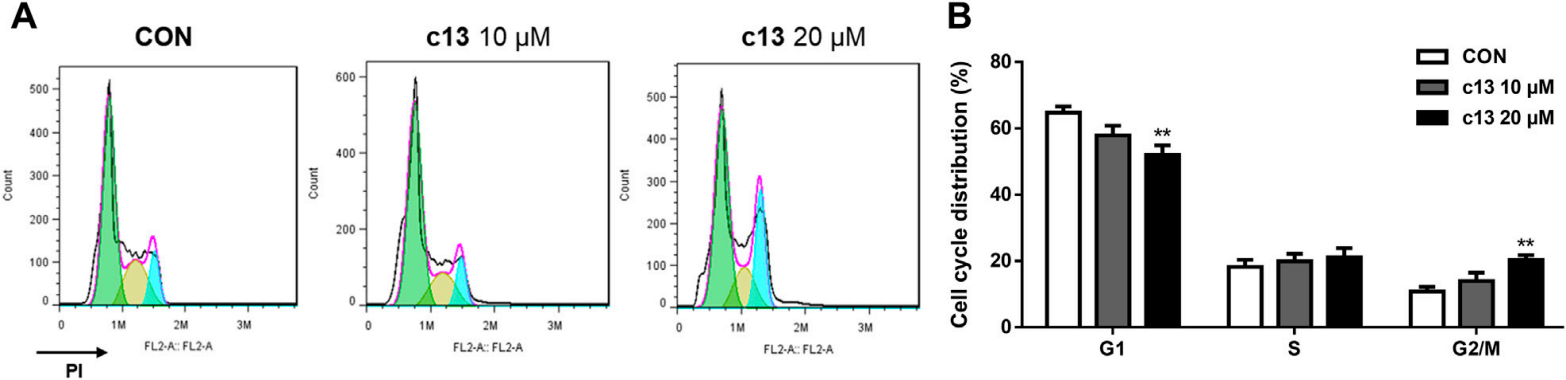
Compound c13 induced cell cycle arrest of Hela cells. **(A)** Cell Cycle were detected by flow cytometry after treatment with compound **c13** at different doses (0, 10, and 20 μM) (n = 6). **(B)** Quantification of the data in **(A)** ^**^ *p* < 0.01 vs. the control group.

To further evaluate the effect of compound **c13** on the cell cycle, the cell cycle distributions of Hela cells after treatment of compound **c13** were detected by flow cytometry. As shown in Figure 3, compound **c13** at 20 μM downregulated the proportion of the G1 phase to 52.05% ± 2.85% of control group. However, after treatment of compound **c13**, the percentage of cells in the G2/M phase was upregulated (Figure 3), which indicated that the cell cycle was blocked in the G2/M stage by compound **c13**. These results suggested that compound **c13** induced G2/M phase block to exert an inhibitory effect on the proliferation of Hela cells.

### 3.3 Compound c13 induced apoptosis in Hela cells

**FIGURE 4:**
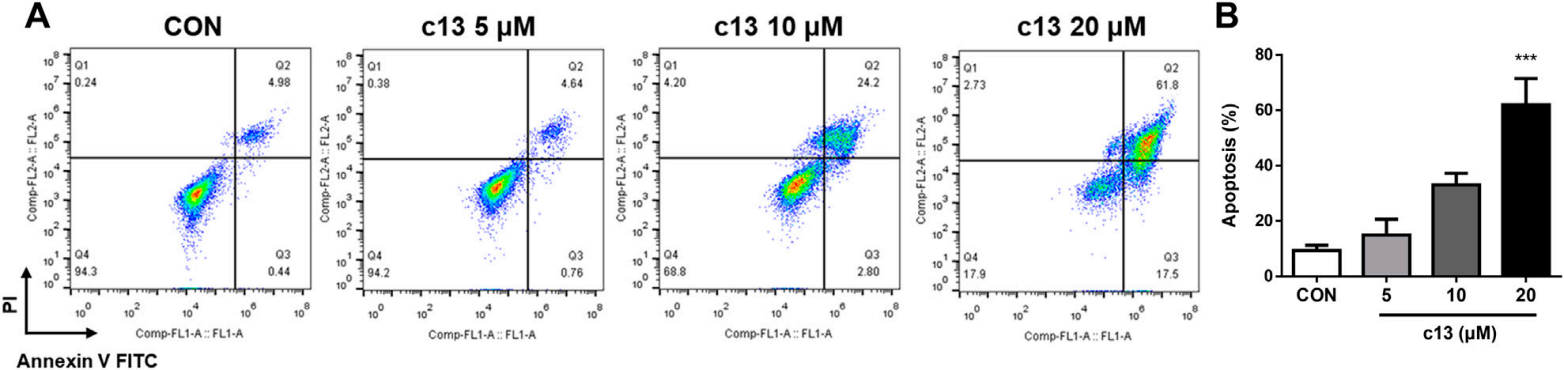
Compound c13 triggered apoptosis of Hela cells. **(A)** Cell apoptosis were detected by flow cytometry after treatment with compound **c13** at different doses (0, 5, 10, and 20 μM) (n = 3). **(B)** Quantification of the data in **(A)** ^***^ *p* < 0.001 vs. the control group.

To assess whether the anti-proliferative effects of compound **c13** on Hela cells were associated with apoptosis, cell apoptosis after treatment of compound **c13** was measured using Annexin V-FITC and propidium iodide (PI) staining by flow cytometer. Compound **c13** concentration-dependently increased the apoptotic rate of Hela cells, with a maximum effect at 20 μM (Figure 4). Compared with the normal control group, compound **c13** at 20 μM markedly raised the apoptotic ratio of Hela cells to 62.03% ± 9.48%. The results indicated that compound **c13** significantly promoted the apoptosis of Hela cells in a concentration-dependent manner.

### 3.4 Compound c13 regulated the expression of apoptosis-associated proteins

**FIGURE 5:**
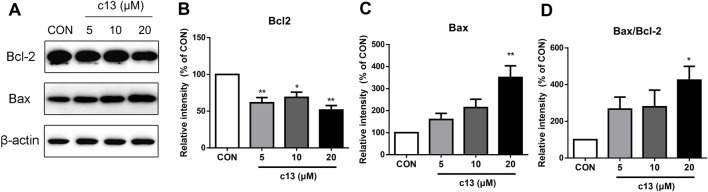
Compound c13 changed pro-apoptotic Bax and anti-apoptotic Bcl-2 protein levels in Hela cells. **(A)** The protein levels of Bax and Bcl-2 were measured by western blot after treatment with compound **c13** at different doses (0, 10, and 20 μM) (n = 3). **(B–D)** Quantification of the data in **(A)** ^*^ *p* < 0.05, ^**^ *p* < 0.01 vs. the control group.

To explore whether the induction of apoptosis caused by compound **c13** was associated with the activation of the apoptosis-related protein, a western blot was used to detect the expression of the protein including Bax and Bcl-2. Consistent with the above data, the results showed that compound **c13** enhanced the protein expression of pro-apoptotic Bax, but diminished the protein level of anti-apoptotic Bcl-2 (Figure 5). Furthermore, the ratio of Bax to Bcl-2 was raised after being treated with compound **c13**, leading to apoptosis in Hela cells.

**FIGURE 6:**
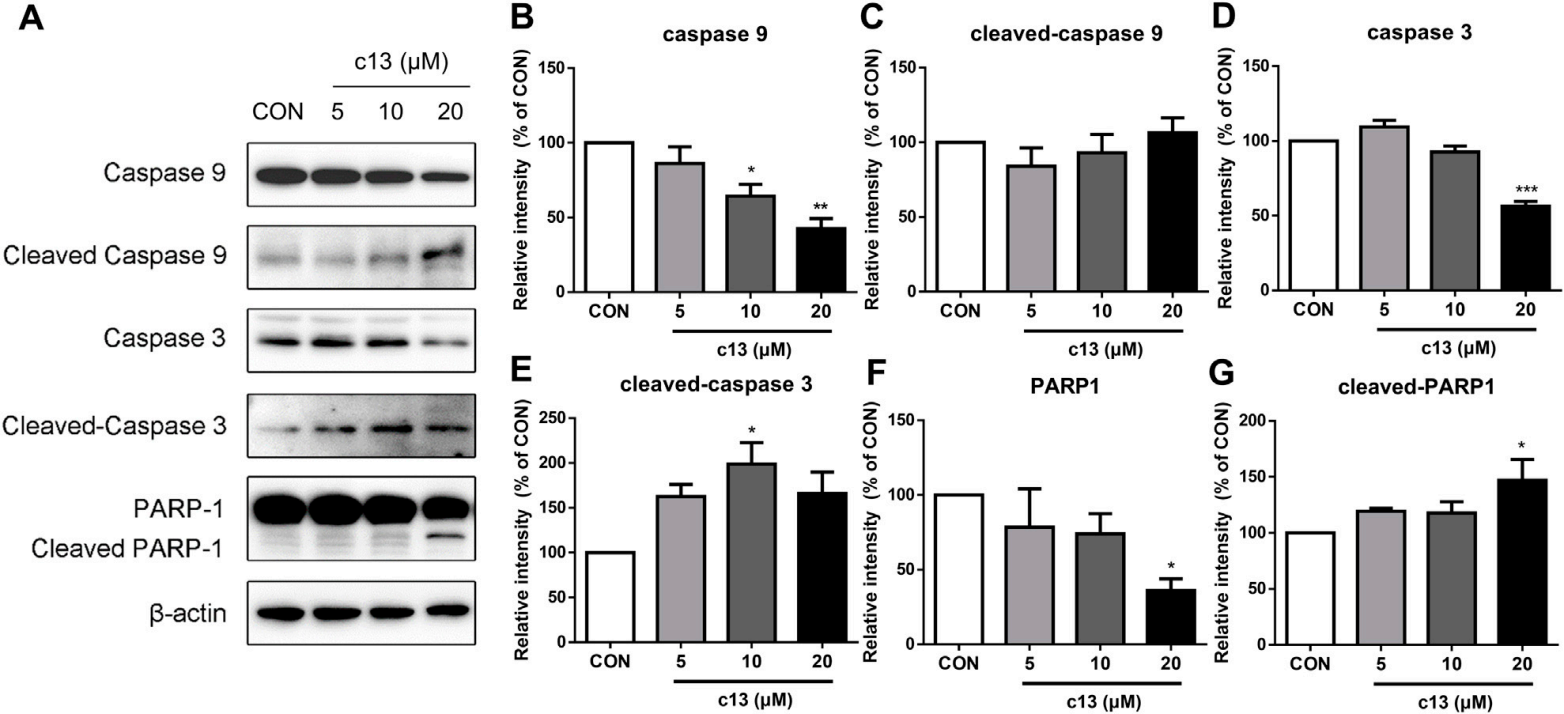
Compound c13 changed the protein expressions of apoptosis signaling pathway in Hela cells. **(A)** The protein levels of apoptosis-associated proteins were measured by western blot after treatment with compound **c13** at different doses (0, 10, and 20 μM) (n = 3). **(B–G)** Quantification of the data in **(A)** ^*^ *p* < 0.05, ^**^ *p* < 0.01, ^***^ *p* < 0.001 vs. the control group.

To determine the potential function of compound **c13** in the regulation of the apoptosis signaling pathway, downstream key proteins containing caspase 3, caspase 9, and PARP1 were detected by western blot. As shown in Figure 6, after treatment of compound **c13** the protein levels of full-length caspase 9, caspase 3, and PARP1 were decreased compared with the control group. Besides, a corresponding increase in the levels of cleaved-caspase 3, and cleaved-PARP1 was observed (Figure 6). However, the level of cleaved-caspase 9 had the tendency of upregulation but had no significant difference. Taken together, these results provided support for the key signaling pathways affected by compound **c13** and indicated that compound **c13** might cause apoptosis of cancer cells through the mitochondrial pathway.

## 4 Discussion

The main cause of cervical cancer is the persistent infection of high-risk human papillomavirus (HPV), which causes almost 100% of cervical cancer cases (De et al., 2015). High-risk-HPV E6/E7 infection could increase the expression of EGFR (Zhang et al., 2022). Currently, the treatment of cervical cancer primarily involves surgery, radiotherapy or chemotherapy based on platinum drugs, which could prolong the survival time of patients to some extent (Burmeister et al., 2022; Abu-Rustum et al., 2023). However, for patients with recurrent or metastatic cervical cancer, there is still a lack of satisfactory and effective treatment options (Cohen et al., 2019). Also, traditional chemotherapy drugs, such as platinum-based agents, have significant toxic side effects. Emerging therapeutic drugs, including targeted molecular drugs and immunotherapeutic drugs for cervical cancer, offer higher efficacy and reduced adverse reactions (Ferrall et al., 2021). Therefore, gefitinib, as one of the first-generation EGFR inhibitors, shows better efficacy against tumor cells with high EGFR expression and has higher specificity for cancer cells than for normal cells (Krishna et al., 2023). In this research, we found that the gefitinib derivative **c13** also demonstrates good safety, selectively inhibiting the proliferation of Hela cervical cancer cells (IC_50_ = 5.66 ± 0.35 μM) with minimal toxicity to HEK293T cells (human embryo kidney epithelial cell) and NIH3T3 cells (mouse embryonic fibroblasts) (IC_50_ > 100 μM).

Considering the promising antitumor efficacy of gefitinib, there is ongoing research on the structural modification of gefitinib. For instance, recent studies have demonstrated that the propyl derivative of gefitinib exhibits potent cytotoxic activity against the A549 cell line (pulmonary adenocarcinoma cell line), A431 (lung cancer cell line), and MDA-MB-231 (breast cancer cell line) by inhibiting EGFR, with IC_50_ values of 10.23 ± 2.3 μM, 10.87 ± 0.18 μM, and 1.68 ± 0.03 μM, respectively (Sharma et al., 2019). Furthermore, our previous findings indicate that most gefitinib structural derivatives possess strong anti-lung cancer activity. The IC_50_ values of compound 4b were 4.42 ± 0.24 μM (NCI-H1299 cells), 3.94 ± 0.01 μM (A549 cells), and 1.56 ± 0.06 μM (NCI-H1437 cells) exerting their anti-tumor effects by triggering apoptosis, increasing the levels of activated caspase 3 and cleaved-PARP, and decreasing the expression of Bcl-2 protein (Liu et al., 2024). Additionally, gefitinib derivatives have shown anti-hepatocellular carcinoma activity, with compounds 11m and 11t demonstrating significant anti-proliferative activity against HepG2 cells, with IC_50_ values of 3.08 ± 0.37 μM and 3.60 ± 0.53 μM, respectively. Mechanistic research suggests that compounds 11m and 11t may induce apoptosis in HepG2 cells through DNA damage (Wu et al., 2024). In the study, compound **c13** exerted antitumor effects by inducing apoptosis in Hela cells.

Apoptosis is an indispensable natural process to eliminate unwanted cells. Caspase 9 is an initiator caspase that acts as a proteolytic signal to activate effector caspases. Effector caspases such as caspase 3 cleave several cellular proteins on their target proteins, such as PARP-1, to promote apoptosis (Langedijk et al., 2015). The activation of the caspase protein family is regulated by the intrinsic death pathway (mitochondrial pathway) or the extrinsic death pathway (surface death receptor pathway). Among them, the mitochondrial pathway is regulated by members of the Bcl-2 family, which mainly consists of two subfamilies: pro-apoptotic proteins (e.g., Bax, Bad, Bim, Bid) and anti-apoptotic proteins (e.g., Bcl-2, Bcl-xI) (Corbett et al., 2012). Abnormal production of apoptotic proteins disrupts the balance between cell proliferation and apoptosis, leading to cancer development (Hubsher et al., 2012). Overexpression of Bax can promote mitochondrial permeability changes, which is accompanied by the activation of the caspase protein family and promotes cell apoptosis through a cascade reaction. In this study, we observed that compound **c13** prominently induced apoptosis in Hela cells using Annexin V-FITC/PI staining by flow cytometer. Further investigations into the underlying mechanism revealed that compound **c13** significantly downregulated the expression of the anti-apoptotic protein Bcl-2, accompanied by a marked upregulation in the expression of the pro-apoptotic protein Bax in Hela cells. Additionally, compound **c13** treatment led to a dose-dependent activation of downstream caspase 3 and PARP-1 cleavage. Collectively, these findings suggest that compound **c13** induces apoptosis in Hela cells through the activation of the mitochondrial pathway.

Investigations have reported that some compounds elicit their anti-cervical cancer properties via inducing apoptosis through the mitochondrial pathway. For instance, Yu Yang et al. delineated that a noval Imidazo [1,2-a] pyridine derivative diminishes Hela cell viability, achieving an IC_50_ of 15.32 μM, by triggering cell apoptosis through the p53/Bax-dependent mitochondrial cascade (Yu et al., 2022). Additionally, Yuqing Huang et al. have demonstrated that β-estradiol imparts notable cytotoxicity to Hela cells, with an IC_50_ of 18.71 ± 1.57 μM after a 72-h treatment period, by instigating mitochondrial apoptosis in HeLa cervical cancer cells, characterized by the dissipation of mitochondrial membrane potential, the activation of caspase enzymes, and a perturbed Bax/Bcl-2 ratio (Huang et al., 2022). Furthermore, Rashmin Khanam et al. have designed and synthesized a series of N-benzhydrylpiperazine derivatives combined with 1,3,4-oxadiazoles (4a-4h) and evaluated their anti-proliferative potential in HeLa cells. Among them, compound 4d, (N-benzhydryl-4-((5-(4-aminophenyl)-1,3,4-oxadiazol-2-yl)methyl)piperazine), exhibited an IC_50_ value of 28.13 ± 0.21 μg/mL and induced Hela cell death through the mitochondrial pathway (Khanam et al., 2019). In this study, we have identified that the gefitinib structural derivative **c13** exhibits robust anti-cervical cancer activity via mitochondrial-mediated apoptosis pathway (IC_50_ = 5.66 ± 0.35 μM) markedly outperforming the parent molecule gefitinib (IC_50_ = 14.18 ± 3.19 μM).

The demise of tumor cells is a multifaceted phenomenon that encompasses a spectrum of factors, notably the generation of reactive oxygen species (ROS), the induction of endoplasmic reticulum (ER) stress, and the activation of autophagy (Gao et al., 2020). Elevated levels of intracellular ROS can lead to oxidative stress, which in turn induces the aggregation of misfolded or unfolded proteins within the ER. This accumulation triggers ER stress and initiates apoptotic pathways in tumor cells (Lin et al., 2019). Furthermore, the activation of autophagy not only promotes apoptosis and inhibits tumorigenesis (Xi et al., 2022), but also mitigates the release of excessive DNA-damaging reactive oxygen species from damaged mitochondria by eliminating these compromised organelles, thereby reducing oncogenic mutations within cells (Bhutia et al., 2013). The anticancer effects of compound **c13** may not only be mediated through the mitochondrial apoptosis pathway but may also involve multiple pathways such as oxidative stress, autophagy, and endoplasmic reticulum stress. Therefore, future studies should explore the potential contributions of these pathways to gain a more comprehensive understanding of the mechanisms underlying the action of compound **c13**.

## 5 Conclusion

In summary, anticancer activities of a series of novel synthetic gefitinib-1,2,3-triazole derivatives on Hela cells were detected. Among all derivatives, compound **c13** exhibited the best inhibitory effect IC_50_ value of 5.66 ± 0.35 μM. Further evaluations including colony formation, cell cycle analysis, and cell apoptosis analysis identified that compound **c13** had good anti-proliferative and pro-apoptotic activity. Western blot analysis provided mechanistic insights, demonstrating that compound **c13** upregulated the Bax/Bcl-2 ratio and increased levels of active caspase 3 and cleaved PARP1, which suggested the involvement of the mitochondrial pathway in compound **c13**-induced apoptosis. The results of the current study underscore the potential of compound **c13** as a promising candidate for the treatment of cervical cancer.

## 6 Experimental

### 6.1 Materials and chemistry

Gefitinib, purchased from Aladdin (Shanghai, China) with a purity ≥99% analyzed by HPLC, was prepared as a stock solution at a concentration of 10 mM in dimethyl sulfoxide (DMSO). Prior to drug administration, the stock solution was diluted with culture medium to achieve the desired working concentrations.

4-(3-((4-Chloro-7-methoxyquinazolin-6-yl)oxy)propyl)morpholine (Compound a, 0.01 mol) was suspended in isopropanol. To this solution, 3-aminophenylacetylene (1.2 g, 0.01 mol) was added. The suspension was stirred at refluxing temperature under a nitrogen atmosphere, during which a solid gradually formed. The reaction progress was monitored by TLC. Upon completion, the reaction mixture was poured into ice water and stirred for 30 min. The resulting solid was collected by filtration and washed with isopropanol to yield compound b, which was then purified to obtain the final product.

Compound **b** and Aryl-azido were added to 200 mL of a water/ t-butanol / THF mixture (1:1:1). The reaction was conducted in the presence of copper sulfate and sodium ascorbate at 80 ℃. Upon completion, as confirmed by TLC, the mixture was extracted with dichloromethane (3 × 100 mL). The combined organic layers were washed successively with water and brine, dried over sodium sulfate, and concentrated *in vacuo*. The residue was purified by column chromatography (V_CH2Cl2_/V_MeOH_ = 40:1) to yield the target compounds c1-14. After numerous experiments, these compounds were accumulated for subsequent experiments.

### 6.2 Biological study

#### 6.2.1 Cell culture

Hela cells were purchased from ATCC (American Type Culture Collection, United States). The cell line was cultured in Dulbecco’s Modified Eagle medium (LifeTech, Grand Island, NY, United States) supplemented with 10% fetal bovine serum (FBS, Siji Green, China), 100 IU/mL of Penicillin and 100 μg/mL Streptomycin in a 5% CO_2_ incubator with a humidified atmosphere at 37°C.

#### 6.2.2 MTT assay

Cell viability was determined using the MTT assay. In brief, Hela cells were seeded in 96-well plates at a density of 3000 cells/well for 24 h. Then, the cells were treated with different concentrations of the investigated compounds for 48 h. After treatment, MTT (Sangon Biotech, Shanghai, China) solution reagent was added into each well to a final concentration of 50 μg/mL, and the cells were incubated for 3 h at 37°C. After the incubation, the formazan product, indicative of viable cells, was solubilized by adding 100 μL of dimethyl sulfoxide (DMSO) to each well. The absorbance of the resulting solution was then measured at 490 nm using a microplate reader (Biotek Instruments, Winooski, VT, United States). The half-maximal inhibitory concentrations (IC50) value was determined by GraphPad Prism 8.0 software.

#### 6.2.3 Plate clone formation assay

Hela cells with a density of 5 × 10^2^ cells/well were seeded in 6-well plates overnight. The cells were treated with different concentrations of compounds (0, 5, 10, and 20 μM) were added and continued to be cultured at 37°C with 5% CO_2_. When more than 50 cell clone formations were visible, the culture medium was discharged. Then, the cells were fixed with 2 mL of 4% paraformaldehyde for 15 min and stained with 2 mL of crystal violet solution at room temperature for 15 min. A microscope was used to take photos and count the number of cell clones.

#### 6.2.4 Apoptosis assay

Hela cells with a density of 1.5 × 10^5^ cells/well were seeded in 12-well plates overnight for adhesion. The cells were treated with different concentrations of compounds (0, 5, 10, and 20 μM) for 48 h. After treatment, the cells were washed with PBS and stained with Annexin V-FITC and Propidium Iodide (PI) for 15 min in the dark. Cell apoptosis rate was detected by BD Accuri C6 Plus flow cytometry (BD Biosciences) and all analyses were performed with FlowJo software.

#### 6.2.5 Cell cycle analysis

Hela cells with a density of 1.5 × 10^5^ cells/well were seeded in 12-well plates overnight. Different concentrations of compounds (0, 5, 10, and 20 μM) were added to treat the cells for 48 h. After treatment, the cells were washed with PBS and then the cells were fixed with 70% ethanol at −20°C overnight. Subsequently, the cells were stained with PI staining solution containing 10 μg/mL RNAase for 30 min in the dark. The data was collected by BD Accuri C6 Plus flow cytometry (BD Biosciences). All analyses were performed with FlowJo software.

#### 6.2.6 Western blot analysis

Whole cell extracts were prepared by lysing cells on ice for 30 min using RIPA lysis buffer (Beyotime Biotechnology, Shanghai, China). Following centrifugation at 12, 000 g for 15 min at 4°C, the supernatants were collected. The protein content concentrations of samples were determined according to the manufacturer’s protocol for the BCA protein assay kit (Thermo Scientific, Rockford, IL, United States).

Equal amounts of protein were resolved using sodium dodecyl sulfate polyacrylamide gel electrophoresis (SDS-PAGE) and subsequently transferred to a 0.2 μm nitrocellulose membrane. The membranes underwent blocking for a minimum of 1 h in 5% skimmed milk diluted in TBST. Subsequently, the blots were incubated overnight at 4°C with primary antibodies. Primary antibodies used were include, Bcl-2 (ABclonal, Wuhan, China), Bax (Wanleibio, Shenyang, China), Caspase 9/p35/p10 antibody (Proteintech, Wuhan, China), Caspase 3/p17/p19 (Proteintech, Wuhan, China), PARP-1 (Proteintech, Wuhan, China), β-actin (Sigma-Aldrich, St Louis, MO, United States). Following incubation, the membranes were washed three times with TBST buffer and then incubated with secondary antibodies (Kangchen Biotechnology, Shanghai, China) for 1 h at room temperature. Subsequently, the blots were washed three times with TBST buffer and developed using an enhanced chemiluminescence reagent (Millipore Corporation, Bedford, MA, United States). The intensity of each protein band was quantitatively analyzed using ImageJ software.

#### 6.2.7 Statistical analysis

All data were statistically analyzed using Graph Prim 8.0 software. The experimental results are all expressed as mean ± Standard Error of Mean (Mean ± SEM). For multiple comparison, data was analyzed by one-way ANOVA followed by Dunnett’s multiple comparisons. And for two groups comparison, student’s t test was used. When P < 0.05, the data is considered statistically significant.

## Data Availability

The original contributions presented in the study are included in the article/Supplementary Material, further inquiries can be directed to the corresponding authors.

## References

[B1] Abu-Rustum N. R. Yashar C. M. Arend R. Barber E. Bradley K. Brooks R. (2023). NCCN Guidelines® insights: cervical cancer, version 1.2024: featured updates to the NCCN guidelines. J. Natl. Compr. Canc Netw. 21 (12), 1224–1233. 10.6004/jnccn.2023.0062 38081139

[B2] Alam M. M. (2022). 1,2,3-Triazole hybrids as anticancer agents: a review. Arch. Pharm. Weinh. 355 (1), e2100158. 10.1002/ardp.202100158 34559414

[B3] Bhutia S. K. Mukhopadhyay S. Sinha N. Das D. N. Panda P. K. Patra S. K. (2013). Autophagy: cancer's friend or foe? Adv. Cancer Res. 118, 61–95. 10.1016/B978-0-12-407173-5.00003-0 23768510 PMC4349374

[B4] Bonandi E. Christodoulou M. S. Fumagalli G. Perdicchia D. Rastelli G. Passarella D. (2017). The 1,2,3-triazole ring as a bioisostere in medicinal chemistry. Drug Discov. Today 22 (10), 1572–1581. 10.1016/j.drudis.2017.05.014 28676407

[B5] Bozorov K. Zhao J. Aisa H. A. (2019). 1,2,3-Triazole-containing hybrids as leads in medicinal chemistry: a recent overview. Bioorg Med. Chem. 27 (16), 3511–3531. 10.1016/j.bmc.2019.07.005 31300317 PMC7185471

[B6] Burmeister C. A. Khan S. F. Schafer G. Mbatani N. Adams T. Moodley J. (2022). Cervical cancer therapies: current challenges and future perspectives. Tumour Virus Res. 13, 200238. 10.1016/j.tvr.2022.200238 35460940 PMC9062473

[B7] Cohen P. A. Jhingran A. Oaknin A. Denny L. (2019). Cervical cancer. Lancet 393 (10167), 169–182. 10.1016/S0140-6736(18)32470-X 30638582

[B8] Corbett A. Pickett J. Burns A. Corcoran J. Dunnett S. B. Edison P. (2012). Drug repositioning for Alzheimer's disease. Nat. Rev. Drug Discov. 11 (11), 833–846. 10.1038/nrd3869 23123941

[B9] De S. Zhou H. DeSantis D. Croniger C. M. Li X. Stark G. R. (2015). Erlotinib protects against LPS-induced endotoxicity because TLR4 needs EGFR to signal. Proc. Natl. Acad. Sci. U. S. A. 112 (31), 9680–9685. 10.1073/pnas.1511794112 26195767 PMC4534288

[B10] Deng C. Yan H. Wang J. Liu K. Liu B. S. Shi Y. M. (2022). 1,2,3-Triazole-containing hybrids with potential antibacterial activity against ESKAPE pathogens. Eur. J. Med. Chem. 244, 114888. 10.1016/j.ejmech.2022.114888 36334453

[B11] Feng L. S. Zheng M. J. Zhao F. L. D. (2021). 1,2,3-Triazole hybrids with anti-HIV-1 activity. Arch. Pharm. Weinh. 354 (1), e2000163. 10.1002/ardp.202000163 32960467

[B12] Ferrall L. Lin K. Y. Roden R. B. S. Hung C. F. Wu T. C. (2021). Cervical cancer immunotherapy: facts and hopes. Clin. Cancer Res. 27 (18), 4953–4973. 10.1158/1078-0432.CCR-20-2833 33888488 PMC8448896

[B13] Gao L. Loveless J. Shay C. Teng Y. (2020). Targeting ROS-mediated crosstalk between autophagy and apoptosis in cancer. Adv. Exp. Med. Biol. 1260, 1–12. 10.1007/978-3-030-42667-5_1 32304028

[B14] Huang Y. Chen S. Lei Y. Chung C. Chan M. Chen L. (2022). β-Estradiol induces mitochondrial apoptosis in cervical cancer through the suppression of AKT/NF-κB signaling pathway. Recent Pat. Anticancer Drug Discov. 17 (3), 312–321. 10.2174/1574892817666211222150409 34951372

[B15] Hubsher G. Haider M. Okun M. S. (2012). Amantadine: the journey from fighting flu to treating Parkinson disease. Neurology 78 (14), 1096–1099. 10.1212/WNL.0b013e31824e8f0d 22474298

[B16] Johnson C. A. James D. Marzan A. Armaos M. (2019). Cervical cancer: an overview of pathophysiology and management. Semin. Oncol. Nurs. 35 (2), 166–174. 10.1016/j.soncn.2019.02.003 30878194

[B17] Jung B. C. Woo S. H. Kim S. H. Kim Y. S. (2024). Gefitinib induces anoikis in cervical cancer cells. BMB Rep. 57 (2), 104–109. 10.5483/BMBRep.2023-0225 38303562 PMC10910092

[B18] Kazandjian D. Blumenthal G. M. Yuan W. He K. Keegan P. Pazdur R. (2016). FDA approval of gefitinib for the treatment of patients with metastatic EGFR mutation-positive non-small cell lung cancer. Clin. Cancer Res. 22 (6), 1307–1312. 10.1158/1078-0432.CCR-15-2266 26980062

[B19] Khanam R. Kumar R. Hejazi I. I. Shahabuddin S. Meena R. Rajamani P. (2019). New N-benzhydrylpiperazine/1,3,4-oxadiazoles conjugates inhibit the proliferation, migration, and induce apoptosis in HeLa cancer cells via oxidative stress-mediated mitochondrial pathway. J. Cell. Biochem. 120 (2), 1651–1666. 10.1002/jcb.27472 30206975

[B20] Krishna A. Sathya M. Mukesh S. Athiyamaan M. S. Banerjee S. Sunny J. (2023). Efficacy and safety of EGFR inhibitor gefitinib in recurrent or metastatic cervical cancer: a preliminary report. Med. Oncol. 40 (7), 203. 10.1007/s12032-023-02070-1 37310466 PMC10264517

[B21] Langedijk J. Mantel-Teeuwisse A. K. Slijkerman D. S. Schutjens M. H. (2015). Drug repositioning and repurposing: terminology and definitions in literature. Drug Discov. Today 20 (8), 1027–1034. 10.1016/j.drudis.2015.05.001 25975957

[B22] Lin Y. Jiang M. Chen W. Zhao T. Wei Y. (2019). Cancer and ER stress: mutual crosstalk between autophagy, oxidative stress and inflammatory response. Biomed. Pharmacother. 118, 109249. 10.1016/j.biopha.2019.109249 31351428

[B23] Liu Z. Liu J. Gao E. Mao L. Hu S. Li S. (2024). Synthesis and *in vitro* antitumor activity evaluation of gefitinib-1,2,3-triazole derivatives. Molecules 29 (4), 837. 10.3390/molecules29040837 38398589 PMC10892142

[B24] Mao L. F. Sun G. Zhao J. Xu G. Q. Yuan M. M. Li Y. M. (2020). Design, synthesis and antitumor activity of icotinib derivatives. Bioorg Chem. 105, 104421. 10.1016/j.bioorg.2020.104421 33181408

[B25] Mao L. F. Wang Z. Z. Wu Q. Chen X. Yang J. X. Wang X. (2022). Design, synthesis, and antitumor activity of erlotinib derivatives. Front. Pharmacol. 13, 849364. 10.3389/fphar.2022.849364 35517789 PMC9065260

[B26] Sharma D. N. Rath G. K. Julka P. K. Gandhi A. K. Jagadesan P. Kumar S. (2013). Role of gefitinib in patients with recurrent or metastatic cervical carcinoma ineligible or refractory to systemic chemotherapy: first study from Asia. Int. J. Gynecol. Cancer 23 (4), 705–709. 10.1097/IGC.0b013e31828b1699 23466569

[B27] Sharma M. J. Kumar M. S. Murahari M. Mayur Y. C. (2019). Synthesis of novel gefitinib-based derivatives and their anticancer activity. Arch. Pharm. Weinh. 352 (5), e1800381. 10.1002/ardp.201800381 31012144

[B28] Sung H. Ferlay J. Siegel R. L. Laversanne M. Soerjomataram I. Jemal A. (2021). Global cancer Statistics 2020: GLOBOCAN estimates of incidence and mortality worldwide for 36 cancers in 185 countries. CA Cancer J. Clin. 71 (3), 209–249. 10.3322/caac.21660 33538338

[B29] Wu J. Huang X. Lu S. Wang Z. Mao L. Li S. (2024). Synthesis of novel gefitinib-conjugated 1,2,3-triazole derivatives and their effect of inducing DNA damage and apoptosis in tumor cells. Molecules 29 (22), 5438. 10.3390/molecules29225438 39598828 PMC11597353

[B30] Xi H. Wang S. Wang B. Hong X. Liu X. Li M. (2022). The role of interaction between autophagy and apoptosis in tumorigenesis (Review). Oncol. Rep. 48 (6), 208. 10.3892/or.2022.8423 36222296 PMC9579747

[B31] Yu Y. Li Y. Yang X. Deng Q. Xu B. Cao H. (2022). A novel imidazo[1,2-a]pyridine compound reduces cell viability and induces apoptosis of HeLa cells by p53/bax-mediated activation of mitochondrial pathway. Anticancer Agents Med. Chem. 22 (6), 1102–1110. 10.2174/1871520621666210805130925 34353269

[B32] Zhang X. Chen C. Ling C. Luo S. Xiong Z. Liu X. (2022). EGFR tyrosine kinase activity and Rab GTPases coordinate EGFR trafficking to regulate macrophage activation in sepsis. Cell. Death Dis. 13 (11), 934. 10.1038/s41419-022-05370-y 36344490 PMC9640671

[B33] Zheng J. Yu J. Yang M. Tang L. (2019). Gefitinib suppresses cervical cancer progression by inhibiting cell cycle progression and epithelial-mesenchymal transition. Exp. Ther. Med. 18 (3), 1823–1830. 10.3892/etm.2019.7754 31410143 PMC6676113

