# Supplementary material for: Anti-cancer activity of synthetic gefitinib-1,2,3-triazole derivatives against Hela cells via induction of apoptosis
Source: Front Chem. 2025 Jan 24;13:1456743. doi: 10.3389/fchem.2025.1456743 (PMC11802567; doi:10.3389/fchem.2025.1456743)

**Supplementary information**

**Anti-cancer activity of synthetic gefitinib-1,2,3-triazole derivatives against Hela cells via induction of apoptosis**

Zhihong Hu^1^, Xixi Hou^2*^, Yongjing Ren^1^, Ziyuan Wu^1^, Dong Yan^1^, Hong Chen^3^ and Lan Wang^1*^

1 College of Basic Medicine and Forensic Medicine, Henan University of Science and Technology, 263 Kaiyuan Road, Luoyang, 471003, China.

2 The First Affiliated Hospital, and College of Clinical Medicine of Henan University of Science and Technology, Luoyang, 471003, China.

3 Luoyang Key Laboratory of Organic Functional Molecules, College of Food and Drug, Luoyang Normal University, Luoyang, 471003, China.

*Corresponding authors:

**Lan Wang**, College of Basic Medicine and Forensic Medicine, Henan University of Science and Technology, 263 Kaiyuan Road, Luoyang 471003, China. E-mail: wldyxdd@163.com

**Xixi Hou**, The First Affiliated Hospital, and College of Clinical Medicine of Henan University of Science and Technology, Luoyang 471003, China. E-mail: lucyfly881104@163.com

**
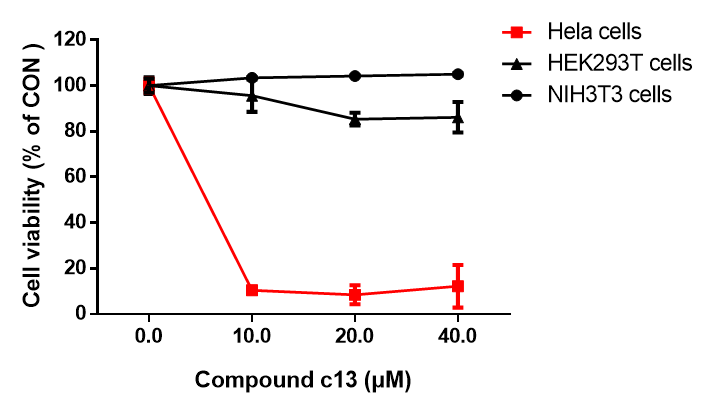
**

Figure S1. The effect of compound **c13** on the survival rate of HEK293T, NIH3T3, and Hela cells. compound **c13** (0, 10, 20, and 40 μM) was used to treat HEK293T, NIH3T3, and Hela cells for 48 hours, and the cell survival rate was detected using the MTT method (n=3).

**Structural Characterization of compound c1-14**

**Compound c1**: HR MS (ESI) m/z: calcd for C31H32ClFN7O3 [M+H]^+^ 604.2239, found 604.2234. ^1^H NMR (400 MHz, DMSO-d_6_) δ 9.60 (s, 1H, NH), 8.69 (s, 1H, CH), 8.49 (s, 1H, CH), 8.27 (t, J = 4.0 Hz, 1H, CH), 7.97-7.87 (m, 2H, Ar-H), 7.69 (m, 1H, Ar-H), 7.59-7.50 (m, 1H, Ar-H), 7.49-7.39 (m, 3H, Ar-H), 7.21 (s, 1H, Ar-H), 5.68 (s, 2H, CH2), 4.21 (t, J = 8.0 Hz, 2H, CH2), 3.95 (s, 3H, OCH3), 3.59 (t, J = 4.6 Hz, 4H, C2H4), 2.49-2.47 (m, 2H, CH2), 2.40-2.39 (m, 4H, C2H4), 2.10-1.88 (m, 2H, CH2). ^13^C NMR (100 MHz, DMSO-d_6_) δ 158.7, 156.8, 156.3, 154.8, 153.3, 148.7, 147.2, 140.5, 134.3, 134.2, 131.2, 131.0, 129.6, 122.4, 122.2, 120.8, 120.3 120.1, 119.3, 117.9, 117.7, 107.7, 103.1, 67.6, 66.6, 56.3, 55.5, 53.9, 52.2, 31.8, 26.3.

**Compound c2**：HR MS (ESI) m/z: calcd for C31H33BrN7O3 [M+H]^+^ 630.1828, found 630.1824. ^1^H NMR (400 MHz, DMSO-d_6_) δ 9.65 (s, 1H, NH), 8.63 (s, 1H, CH), 8.49 (s, 1H, CH), 8.29 (s, 1H, Ar-H), 8.06-7.83 (m, 2H, Ar-H), 7.72 (d, J = 8.0 Hz, 1H, Ar-H), 7.62-7.54 (m, 1H, Ar-H), 7.51-7.39 (m, 2H, Ar-H), 7.39-7.29 (m, 1H, Ar-H), 7.28-7.17 (m, 2H, Ar-H), 5.76 (s, 2H, CH2), 4.25 (t, J = 6.4 Hz, 2H, CH2), 3.95 (s, 3H, OCH3), 3.75-3.62 (m, 4H, C2H4), 2.97-2.66 (m, 5H), 2.26-2.03 (m, 2H, CH2). ^13^C NMR (100 MHz, DMSO-d_6_) δ 156.9, 154.8, 153.3, 148.5, 147.3, 146.9, 140.5, 135.3, 133.4, 131.3, 131.0, 130.9, 129.5, 128.8, 123.4, 122.5, 122.4, 120.9, 119.4, 109.4, 107.8, 103.5, 67.4, 65.5, 56.3, 55.0, 53.6, 53.0, 25.1.

**Compound c3**: HR MS (ESI) m/z: calcd for C31H33FN7O3 [M + H]^+^ 570.2629, found 570.2625. ^1^H NMR (400 MHz, DMSO-d_6_) δ 9.64 (s, 1H, NH), 8.74 (s, 1H, CH), 8.53 (s, 1H, CH), 8.32 (s, 1H, Ar-H), 7.95 (d, J = 8.0 Hz, 2H, Ar-H), 7.61 (d, J = 8.0 Hz, 1H, Ar-H), 7.52-7.47 (m, 2H, Ar-H), 7.30-7.18 (m, 5H, Ar-H), 5.74 (s, 2H, CH2), 4.24 (t, J = 6.3 Hz, 2H, CH2), 3.98 (s, 3H, OCH3), 3.63-3.56 (m, 4H, C2H4), 2.55-2.41 (m, 5H), 2.08-2.01(m, 2H, CH2). ^13^C NMR (100 MHz, DMSO-d_6_) δ 163.9, 161.4, 156.8, 154.8, 153.3, 148.7, 147.4, 147.2, 140.6, 139.1, 131.4, 129.5, 124.5, 124.4, 122.4, 122.3, 120.8, 119.3, 115.6, 115.5, 115.2, 109.4, 107.7, 103.1, 67.6, 66.6, 56.3, 55.4, 53.9, 52.8, 26.3.

**Compound c4**: HR MS (ESI) m/z: calcd for C31H32BrFN7O3 [M+H]^+^ 648.1734 found 648.1727. ^1^H NMR (400 MHz, DMSO-d_6_) δ 9.62 (s, 1H), 8.64 (s, 1H, 8.26 (s, 1H), 8.15-7.90 (m, 2H), 7.77 (s, 1H), 7.59-7.46 (m, 3H), 7.27-7.18 (m, 2H), 5.75 (s, 2H), 4.49-4.23 (m, 3H), 3.98 (s, 3H), 3.59 (m, 4H), 2.51 (m, 5H), 2.03-2.01 (m, 2H). ^13^C NMR (100 MHz, DMSO-d_6_) δ 163.14, 160.70, 154.65, 148.88, 146.96, 140.53, 137.44, 137.36, 135.24, 135.16, 131.25, 129.50, 122.56, 120.91, 119.42, 118.42, 118.18, 117.97, 103.55, 67.62, 66.63, 56.39, 55.44, 53.89, 53.38, 26.32.

**Compound c5**: HR MS (ESI) m/z: calcd for C31H32BrFN7O3 [M+H]^+^ 648.1734 found 648.1726. ^1^H NMR (400 MHz, DMSO-d_6_ δ 9.60 (s, 1H), 8.64 (s, 1H), 8.26 (s, 1H), 8.11-7.90 (m, 2H), 7.66-7.36 (m, 6H), 5.71 (s, 2H), 4.27-4.16 (m, 3H), 4.02-3.92 (m, 3H), 3.65-3.54 (m, 4H), 2.58-2.51 (m, 5H), 2.07-2.00 (m, 2H). ^13^C NMR (100 MHz, DMSO-d_6_) δ 161.79, 159.29, 154.77, 148.80, 147.05, 140.55, 132.86, 132.82, 131.24, 129.48, 128.59, 128.56, 122.92, 122.76, 122.66, 122.46, 122.25, 120.83, 119.74, 119.49, 119.34, 103.38, 67.62, 66.62, 56.35, 55.44, 53.88, 47.19, 47.16, 26.31.

**Compound c6**: HR MS (ESI) m/z: calcd for C31H32ClFN7O3 [M+H]^+^ 604.2239, found 604.2232. ^1^H NMR (400 MHz, DMSO-d_6_) δ 9.60 (s, 1H), 8.61 (s, 1H), 8.25 (s, 1H), 8.08 – 7.88 (m, 2H), 7.57-7.29 (m, 6H), 5.75 (s, 2H), 4.27-4.15 (m, 3H), 4.03-3.91 (m, 3H), 3.65-3.54 (m, 5H), 2.55-2.51 (m, 5H), 2.02-1.99 (m, 2H). ^13^C NMR (100 MHz, DMSO-d_6_) δ 161.13, 154.75, 148.81, 146.92, 140.52, 133.07, 132.98, 131.27, 130.09, 129.48, 122.50, 122.36, 120.89, 119.39, 117.68, 117.43, 115.56, 115.35, 67.62, 66.61, 56.36, 55.43, 53.87, 50.72, 26.30.

**Compound c7**: HR MS (ESI) m/z: calcd for C31H31ClFN7O3 [M+H]^+^ 604.2239, found 604.2232. ^1^H NMR (400 MHz, DMSO-d_6_) δ 9.62 (s, 1H), 8.62 (s, 1H), 8.23-7.89 (m, 3H), 7.73-7.35 (m, 6H), 5.78 (s, 2H), 4.46-4.21 (m, 3H), 4.02-3.89 (m, 3H), 3.72-3.55 (m, 5H), 2.57-2.51 (m, 5H), 2.03-2.00 (m, 2H). ^13^C NMR (100 MHz, DMSO-d_6_) δ 163.11, 160.62, 156.37, 148.86, 146.69, 140.48, 135.50, 135.45, 132.39, 132.29, 131.25, 129.45, 129.13, 126.39, 126.36, 122.57, 122.32, 120.96, 115.60, 115.38, 103.57, 67.60, 66.56, 56.38, 55.41, 53.83, 45.09, 30.47, 26.25.

**Compound c8**: HR MS (ESI) m/z: calcd for C31H33N8O5 [M+H]^+^ 597.2574, found 597.2566. ^1^H NMR (400 MHz, DMSO-d_6_) δ 9.62 (s, 1H), 8.64 (s, 1H), 8.28 (s, 1H), 8.18 (d, J=8.0Hz, 1H), 8.08-7.90 (m, 1H), 7.78 (t, J=8.0Hz, 1H), 7.66 (t, J=8.0Hz, 1H), 7.58 (d, J=8.0Hz, 1H), 7.47 (t, J=4.0Hz, 1H), 7.19 (t, J=8.0Hz, 1H), 6.04 (s, 2H), 4.25-3.97 (m, 5H), 3.64-3.51 (m, 5H), 2.55-2.51 (m, 5H), 2.02-1.99 (m, 2H). ^13^C NMR (100 MHz, DMSO-d_6_) δ 154.70, 148.83, 148.08, 147.03, 140.57, 134.93, 131.23, 131.18, 130.75, 130.20, 129.52, 125.52, 122.62, 122.87, 122.48, 120.85, 119.37, .103.44, 67.62, 66.61, 56.36, 55.43, 55.37, 53.87, 50.69, 26.30.

**Compound c9**: HR MS (ESI) m/z: calcd for C31H33N8O5 [M+H]^+^ 597.2574, found 597.2567. ^1^H NMR (400 MHz, DMSO-d_6_) δ 9.63 (s, 1H), 8.72 (s, 1H), 8.28-8.26 (m, 3H), 7.98-7.89 (m, 2H), 7.61-7.43 (m, 5H), 5.86 (s, 2H), 4.34-4.16 (m, 3H), 3.95 (s, 3H), 3.63-3.38 (m, 5H), 2.56-2.51 (m, 5H), 2.03-2.00 (m, 2H). ^13^C NMR (100 MHz, DMSO-d_6_) δ 156.80, 154.86, 148.77, 147.75, 147.25, 143.86, 140.49, 135.94, 131.91, 131.45, 131.22, 129.52, 129.35, 124.44, 122.55, 120.50, 120.89, 119.41, 103.24, 67.61, 66.55, 56.34, 55.41, 53.83, 52.63, 49.38, 26.24.

**Compound c10**: HR MS (ESI) m/z: calcd for C31H33N8O6 [M+H]^+^ 613.2523, found 613.3378. ^1^H NMR (400 MHz, DMSO-d_6_) δ 9.65 (s, 1H), 9.11 (s, 1H), 8.37 (s, 1H), 8.12-7.94 (m, 5H), 7.71-7.50 (m, 3H), 4.24-4.21 (m, 2H), 4.08 (s, 3H), 3.96 (s, 3H), 3.65-3.54 (m, 5H), 2.56-2.51 (m, 5H), 2.05-1.97 (m, 2H). ^13^C NMR (100 MHz, DMSO-d_6_) δ 152.07, 148.80, 148.73, 147.16, 140.65, 131.03, 130.74, 129.59, 126.54, 123.77, 119.60, 116.76, 108.84, 107.17, 104.17, 103.36, 97.04, 67.63, 66.62, 57.68, 56.35, 55.44, 53.89, 31.17, 29.48, 26.32

**Compound c11**: HR MS (ESI) m/z: calcd for C31H33BrN7O3 [M+H]^+^ 630.1828, found 630.1819. ^1^H NMR (400 MHz, DMSO-d_6_) δ 9.59 (s, 1H, NH), 8.65 (s, 1H, CH), 8.25 (d, J = 1.9 Hz, 1H, CH), 7.97-7.85 (m, 2H, Ar-H), 7.65-7.58 (m, 2H, Ar-H), 7.57-7.52 (m, 1H, Ar-H), 7.48-7.42 (m, 1H, Ar-H), 7.37-7.29 (m, 2H, Ar-H), 5.66 (s, 2H, CH2), 4.21 (t, J = 6.3 Hz, 2H, CH2), 3.95 (s, 3H, OCH3), 3.59 (t, J = 4.6 Hz, 6H, C2H4, CH2), 2.45-2.37 (m, 4H, C2H4), 2.07-1.95 (m, 2H, CH2). ^13^C NMR (100 MHz, DMSO-d_6_) δ 156.7, 154.8, 148.8, 147.2, 140.5, 135.9, 132.2, 131.3, 130.7, 129.5, 122.4, 122.2, 121.9, 120.8, 119.3, 103.3, 67.6, 66.6, 56.3, 55.4, 53.8, 52.8, 31.7, 29.5, 29.0, 27.0, 26.2.

**Compound c12**:HR MS (ESI) m/z: calcd for C32H33N8O3 [M+H]^+^ 577.2676, found 577.2671. ^1^H NMR (400 MHz, DMSO-d_6_) δ 9.60 (s, 1H, NH), 8.69 (s, 1H, CH), 8.50 (s, 1H, CH), 8.27 (t, J = 1.9 Hz, 1H, Ar-H), 8.00 – 7.87 (m, 3H, Ar-H), 7.84-7.72 (m, 1H, Ar-H), 7.66- 7.37 (m, 4H, Ar-H), 7.22 (s, 1H, Ar-H), 5.89 (s, 2H, CH2), 4.21 (t, J = 6.3 Hz, 2H, CH2), 3.95(s, 3H, OCH3), 3.61-3.58 (m, 4H, C2H4), 2.51-2.50 (m, 6H, CH2, C2H4), 2.05-2.01 (m, 2H, CH2). ^13^C NMR (100 MHz, DMSO-d_6_) δ 156.8, 154.8, 150.0, 148.7, 147.1, 140.6, 139.2, 134.4, 133.9, 131.2, 129.7, 129.6, 129.5, 122.6, 122.5, 120.8, 119.4, 117.5, 116.0, 111.7, 103.2, 71.0, 67.6, 66.6, 56.3, 55.4, 55.3, 53.8, 51.7, 26.2.

**Compound c13**: HR MS (ESI) m/z: calcd for C31H32Br2N7O3 [M+H]^+^ 708.0933, found 708.0931. ^1^H NMR (400 MHz, DMSO-d_6_) δ 9.61 (s, 1H, NH), 8.71 (s, 1H, CH), 8.27 (s, 1H, CH), 8.06 (s, 1H, Ar-H), 7.94-7.87 (m, 2H, Ar-H), 7.86 (s, 1H, Ar-H), 7.70-7.61 (m, 2H, Ar-H), 7.57 (d, J = 8.0 Hz, 1H, Ar-H), 7.46 (t, J = 8.0 Hz, 1H, Ar-H), 5.69 (s, 2H, CH2), 4.23-4.20 (m, 3H, CH, CH2), 4.00-3.87 (m, 3H, OCH3), 3.59 (t, J = 4.6 Hz, 4H, C2H4), 2.60-2.47 (m, 6H, CH2, C2H4), 2.03-1.99 (m, 2H, CH2). ^13^C NMR (100 MHz, DMSO-d_6_) δ 154.7, 148.8, 147.2, 147.0, 140.8, 140.6, 133.7, 131.2, 130.7, 129.5, 123.2, 122.5, 122.4, 120.8, 119.4, 104.6, 103.5, 92.2, 67.6, 66.6, 56.4, 55.4, 53.9, 52.0, 26.3.

**Compound c14**: HR MS (ESI) m/z: calcd for C32H36N7O3 [M + H]^+^ 566.2880, found 566.2873. ^1^H NMR (400 MHz, DMSO-d_6_) δ 9.60 (s, 1H, NH), 8.62 (s, 1H, CH), 8.25 (s, 1H, CH), 8.13-8.04 (m, 1H, Ar-H), 7.90 (d, J = 8.0 Hz, 1H, Ar-H), 7.74-7.65 (m, 1H, Ar-H), 7.55 (d, J = 8.0 Hz, 1H, Ar-H), 7.44 (t, J = 8.0 Hz, 1H, Ar-H), 7.35-7.14 (m, 5H, Ar-H), 5.60 (s, 2H, CH2), 4.22-4.19 (m, 2H, CH2), 3.98 (s, 3H, OCH3), 3.63-3.54 (m, 4H, C2H4), 2.52-2.50 (m, 4H, C2H4), 2.32-2.30 (m, 1H, CH2), 2.29 (s, 3H, CH3), 2.03-2.00 (m, 2H, CH2). ^13^C NMR (100 MHz, DMSO-d_6_) δ 148.9, 147.1, 140.5, 138.0, 133.5, 131.4, 129.8, 129.7, 129.5, 129.1, 129.0, 128.5, 128.4, 122.4, 122.0, 120.8, 119.3, 67.6, 66.6, 65.5, 56.4, 55.4, 53.8, 53.3, 30.5, 26.3, 21.2, 19.1, 14.0.

^1^H NMR and ^13^C NMR spectrums of compound **c1**


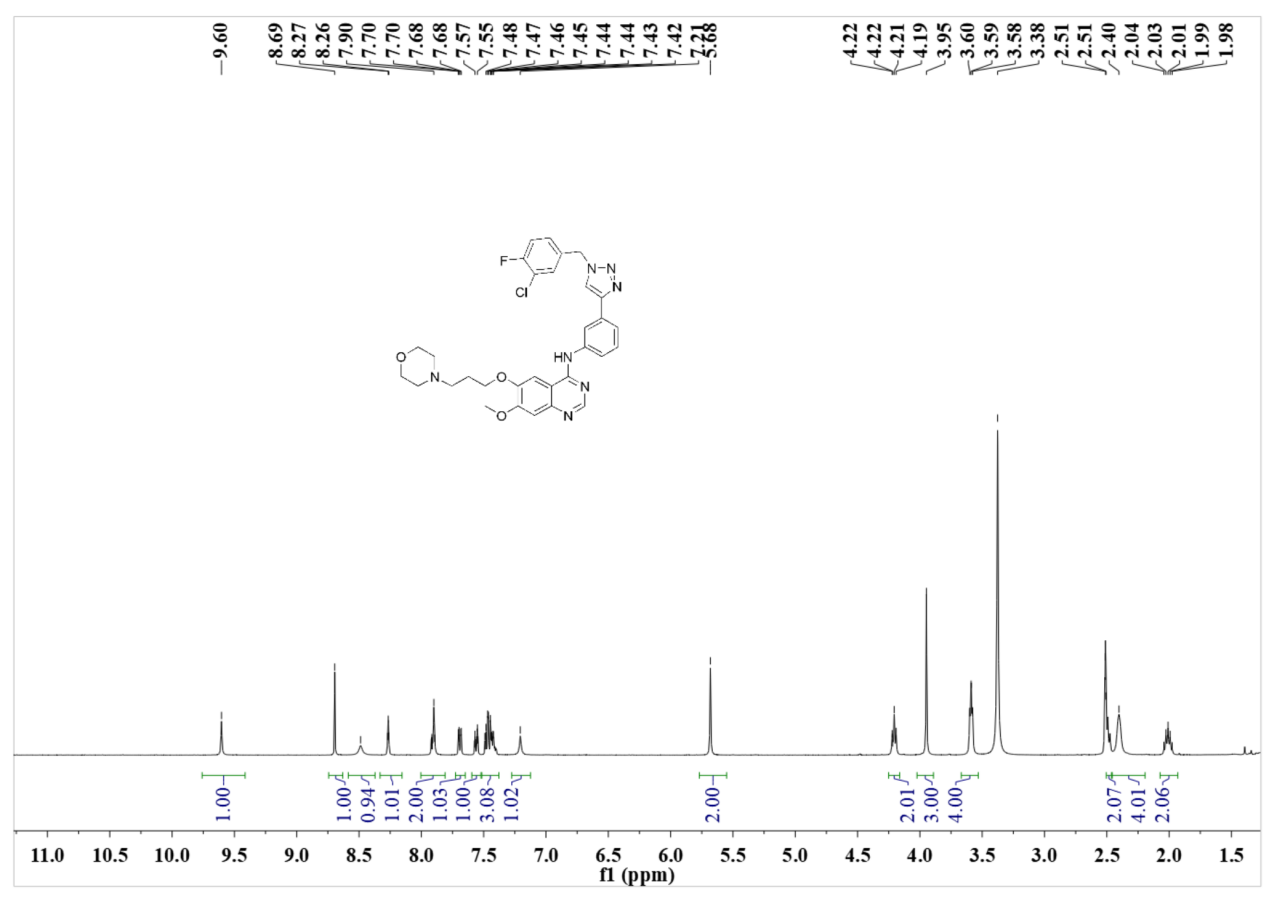


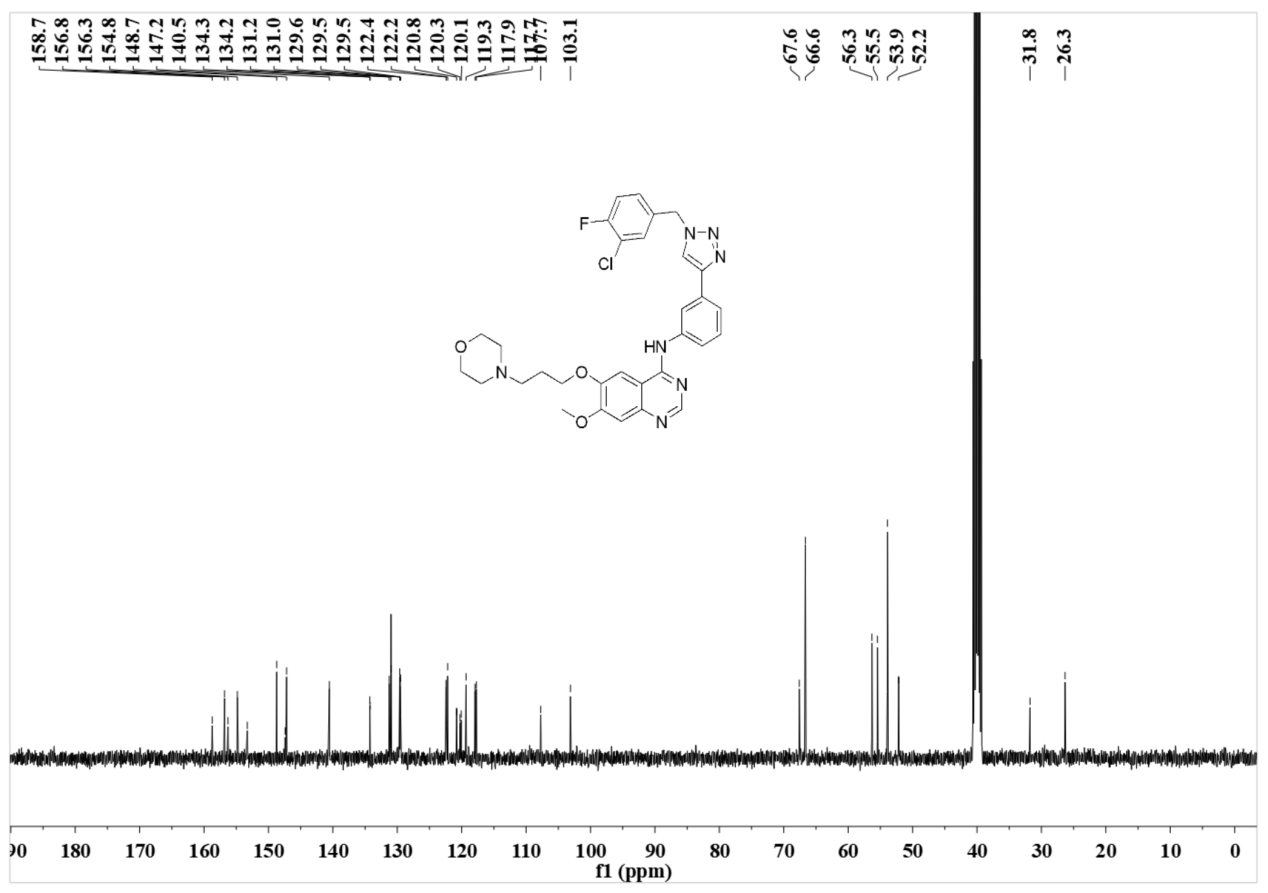


^1^H NMR and ^13^C NMR spectrums of compound **c2**


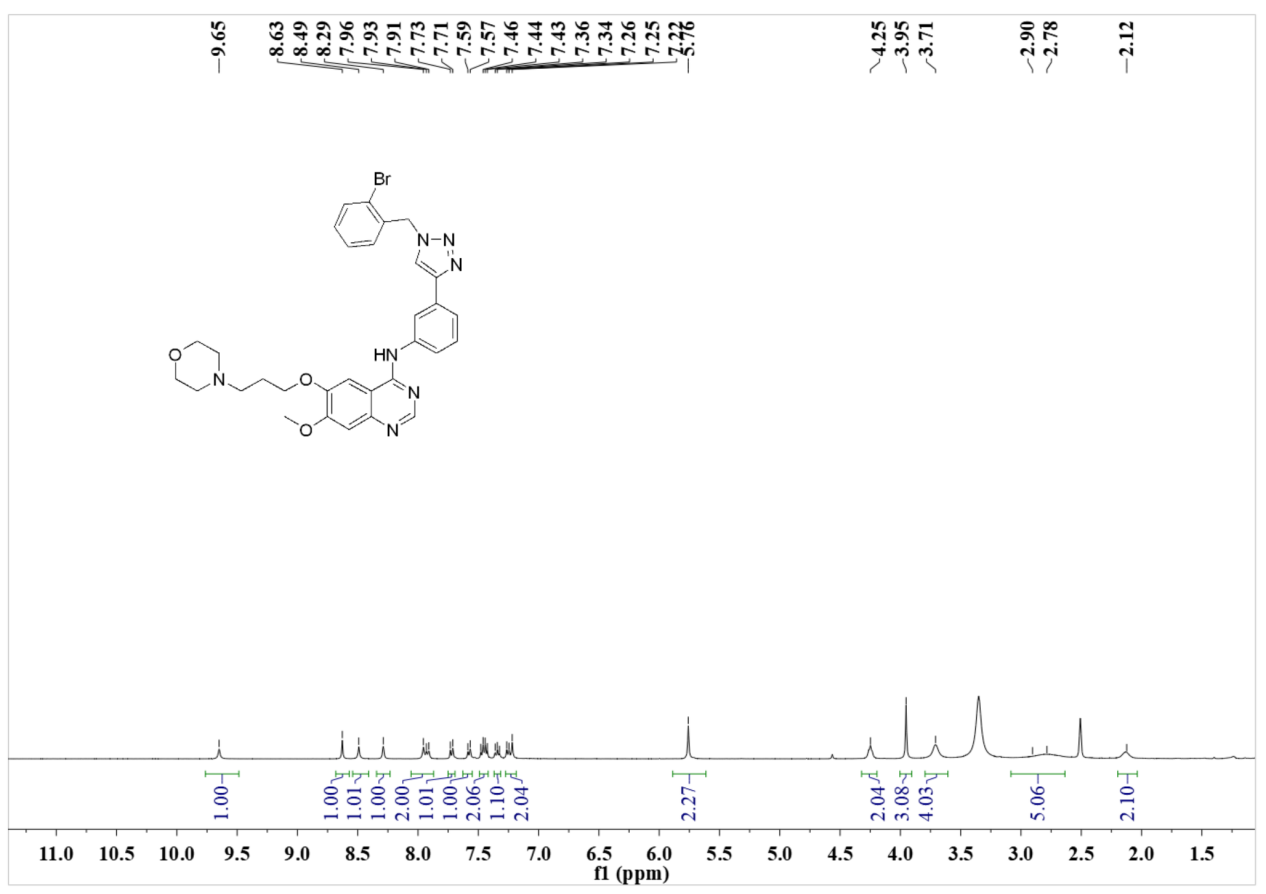


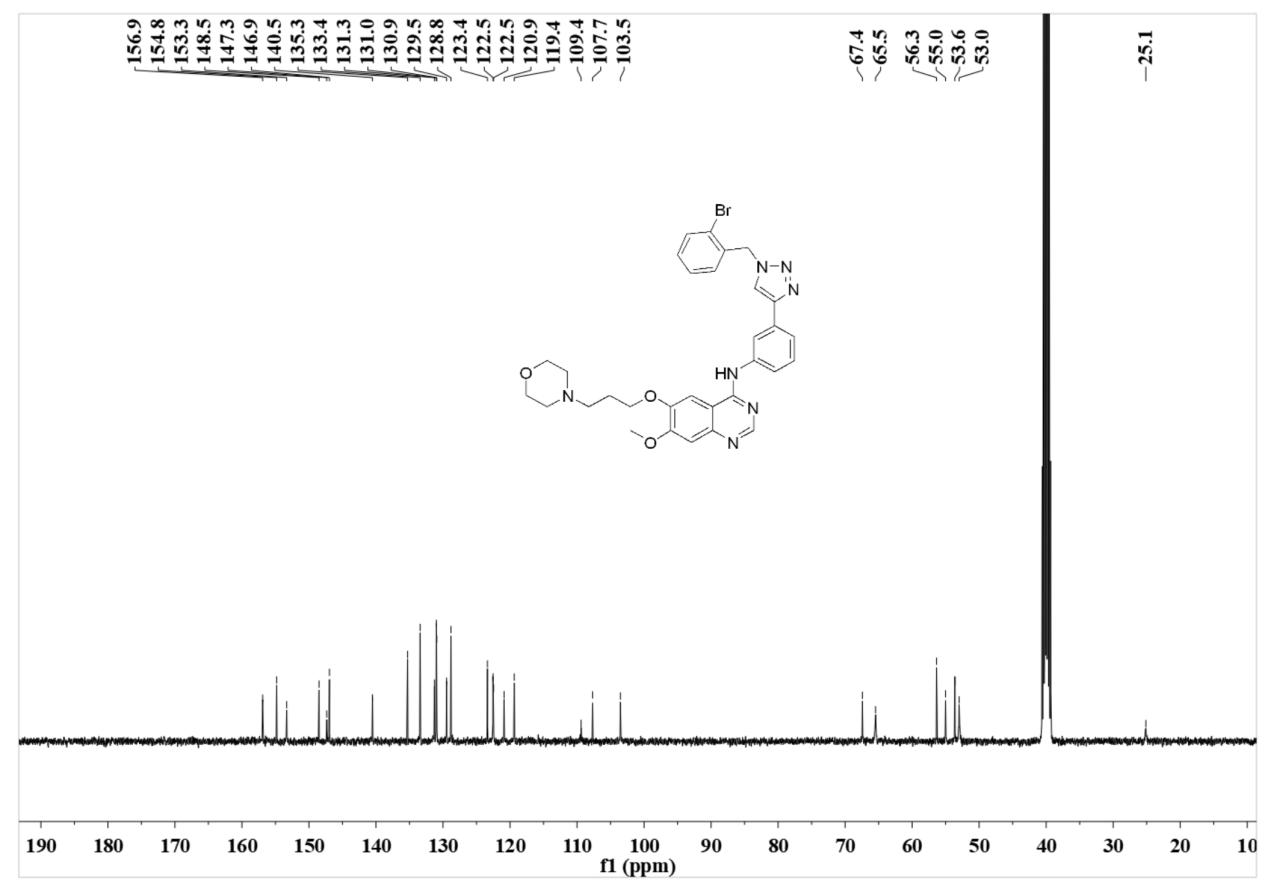


^1^H NMR and ^13^C NMR spectrums of compound **c3**


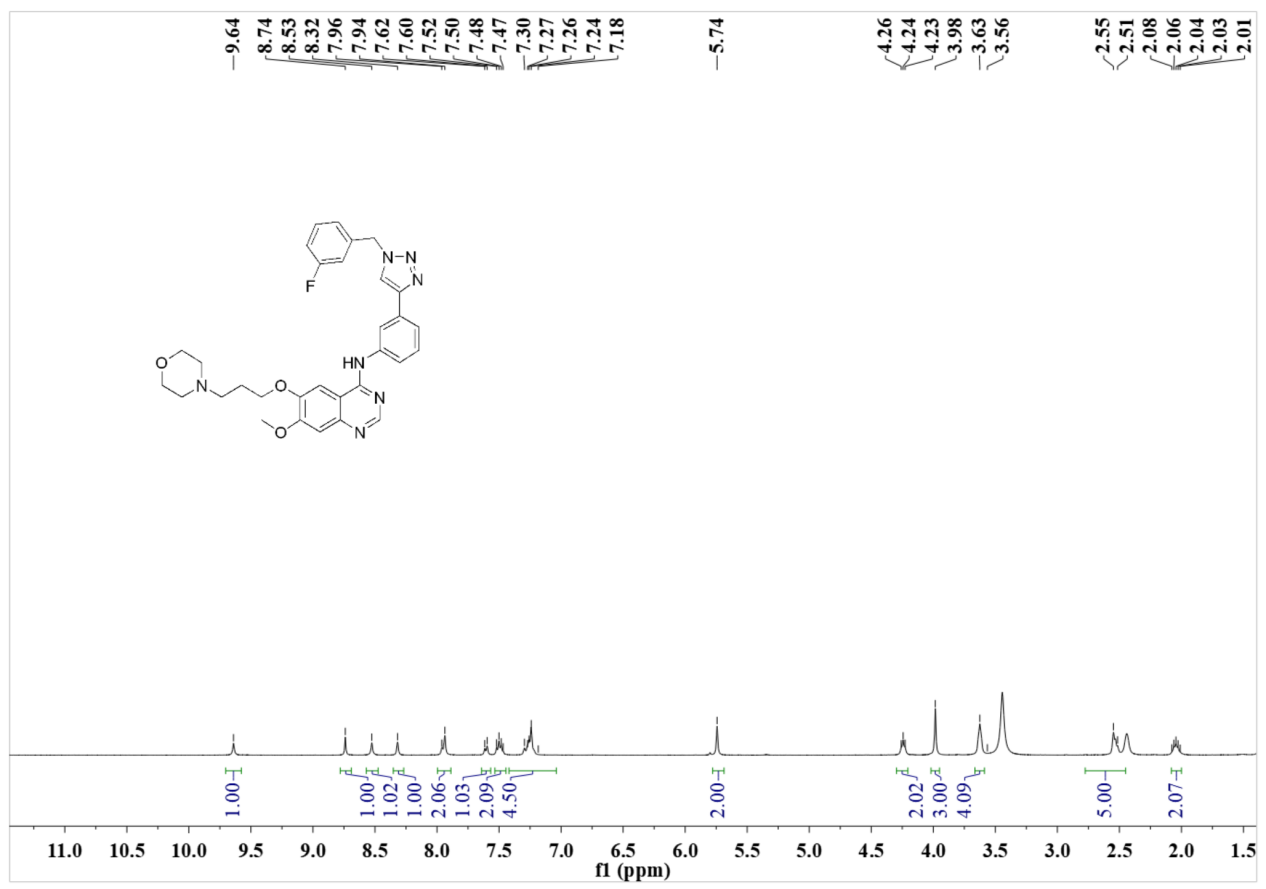


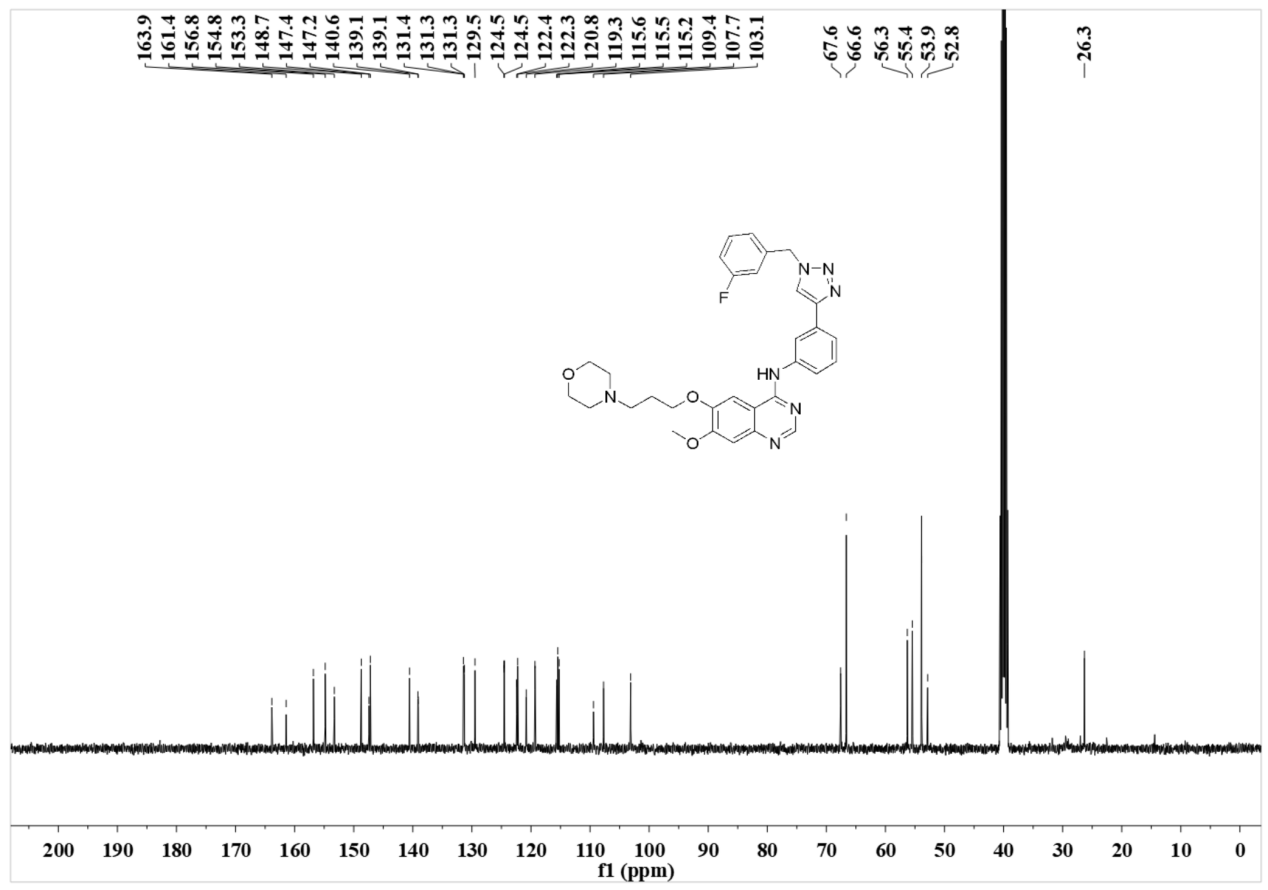


^1^H NMR and ^13^C NMR spectrums of compound **c4**


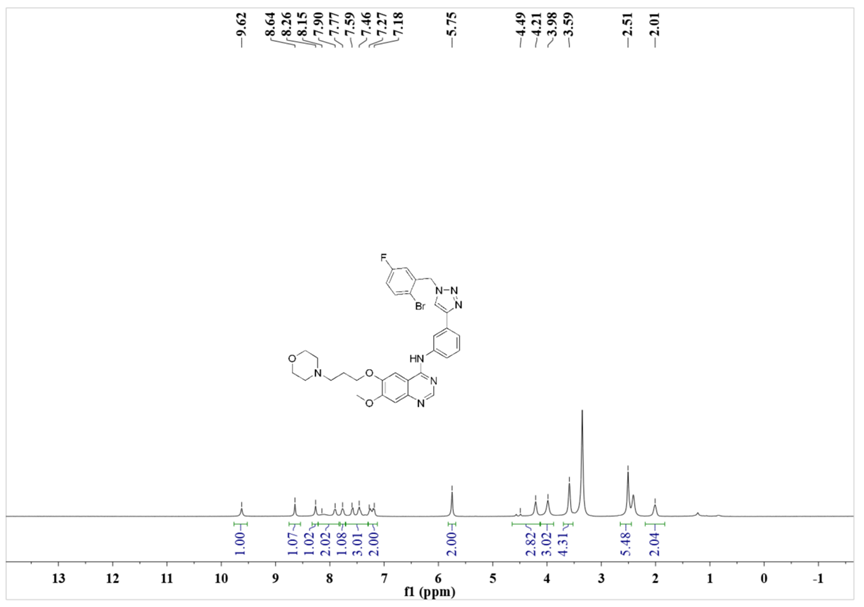


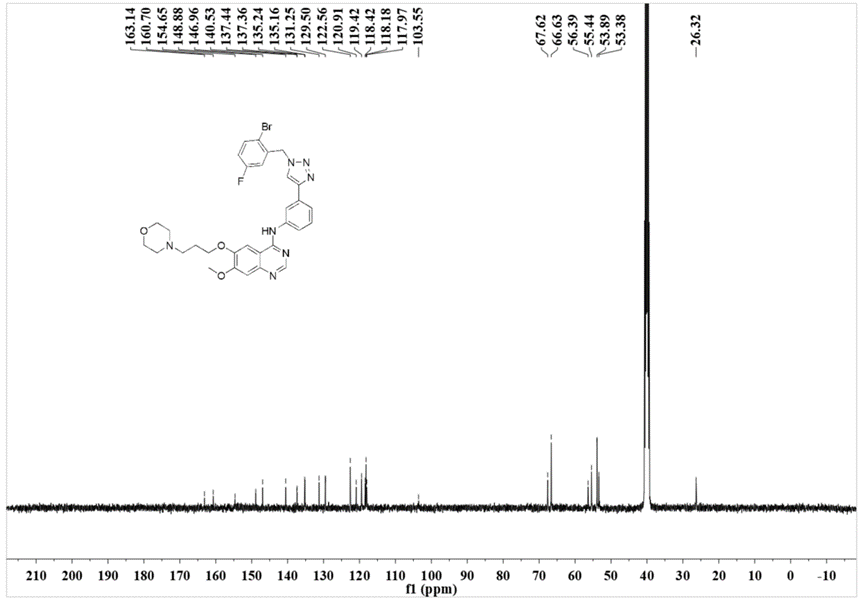


^1^H NMR and ^13^C NMR spectrums of compound **c5**


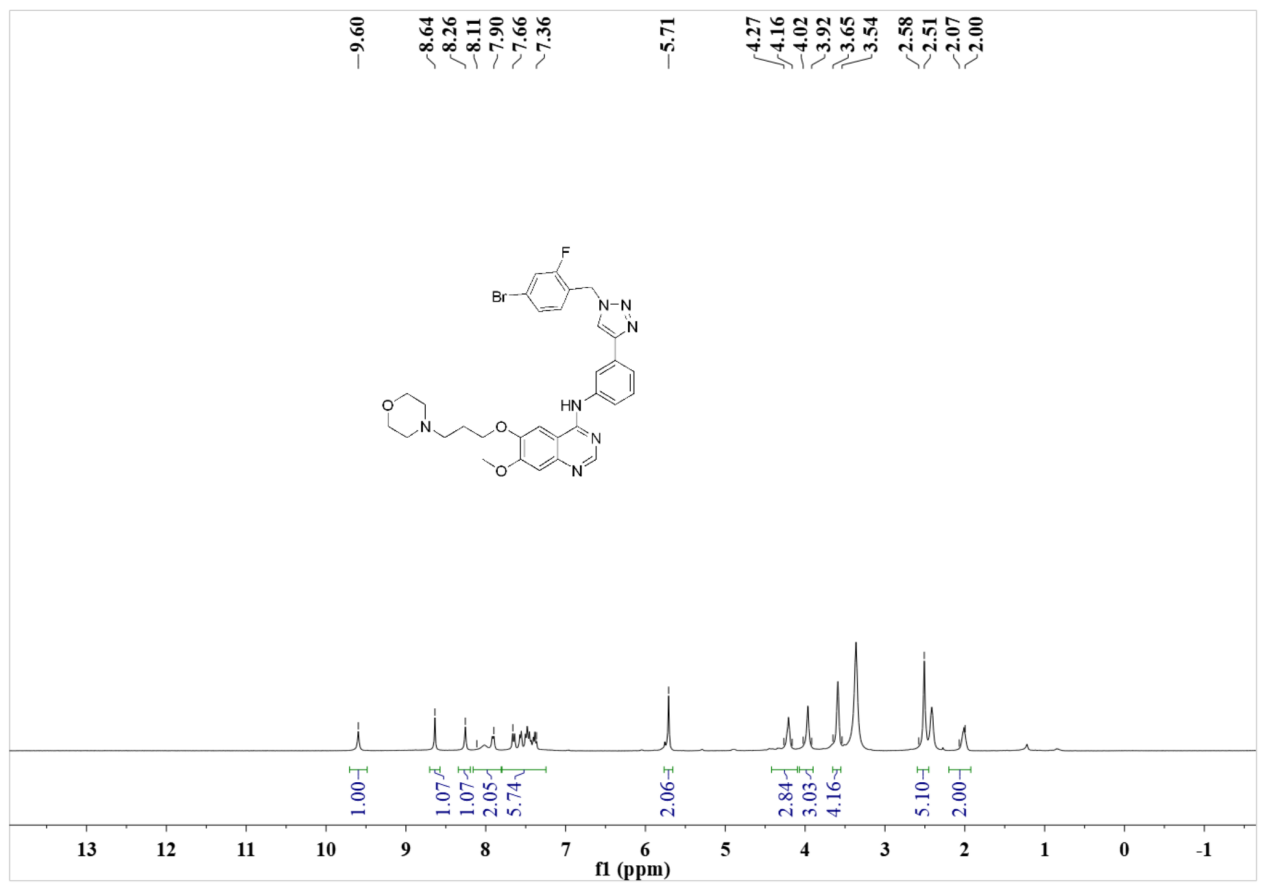


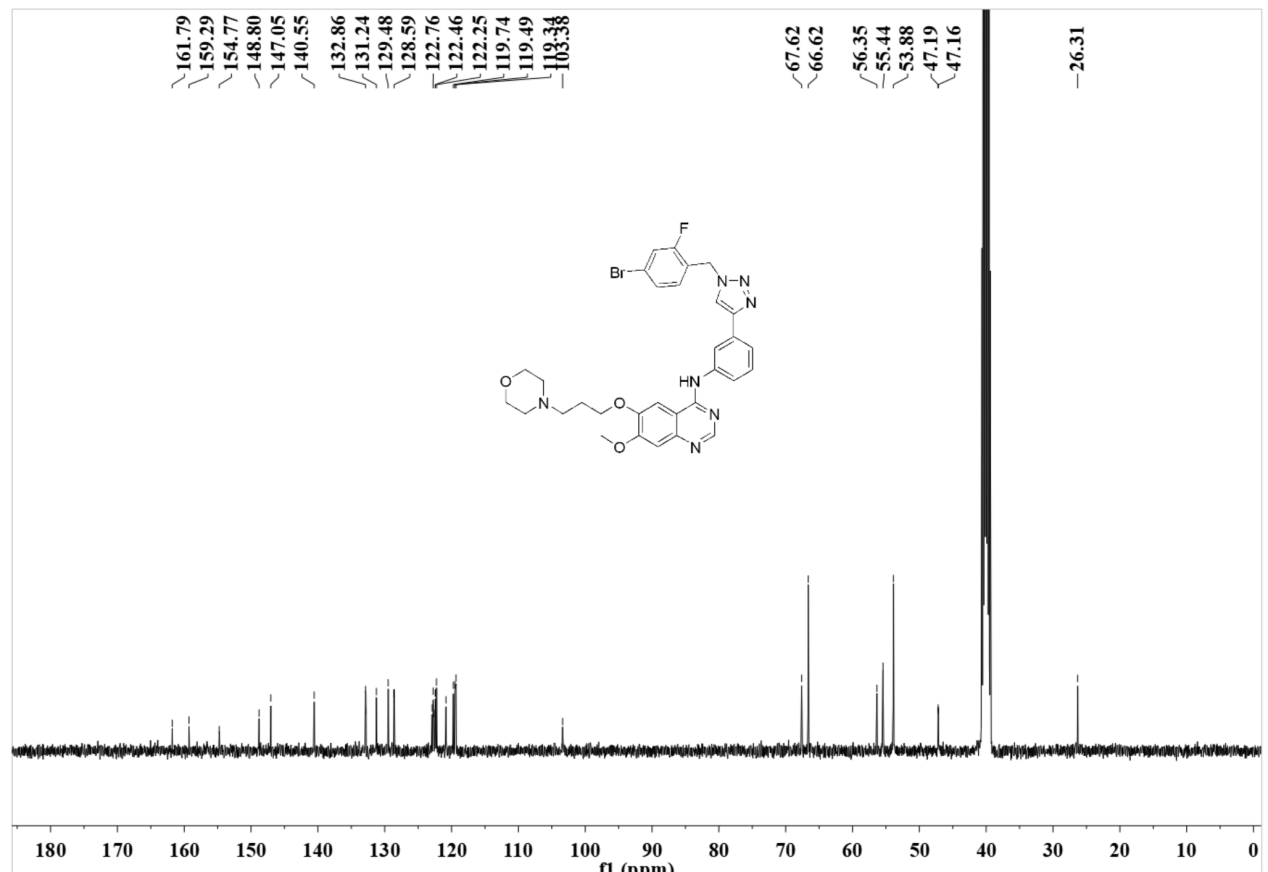


^1^H NMR and ^13^C NMR spectrums of compound **c6**


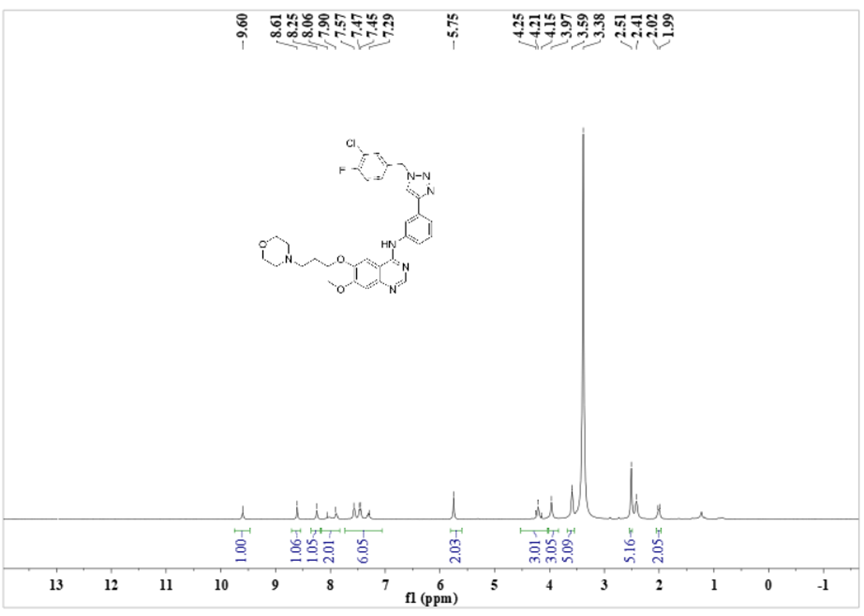


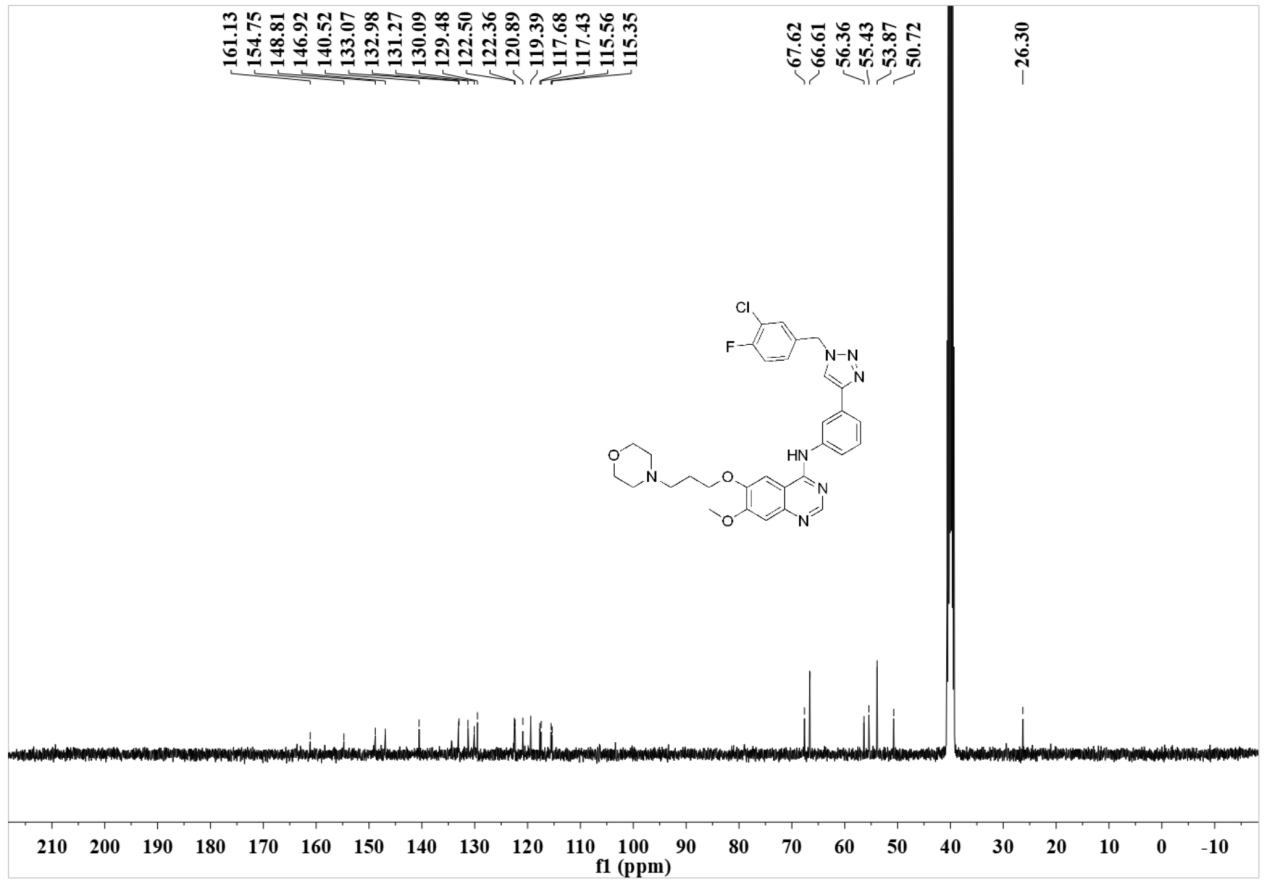


^1^H NMR and ^13^C NMR spectrums of compound **c7**


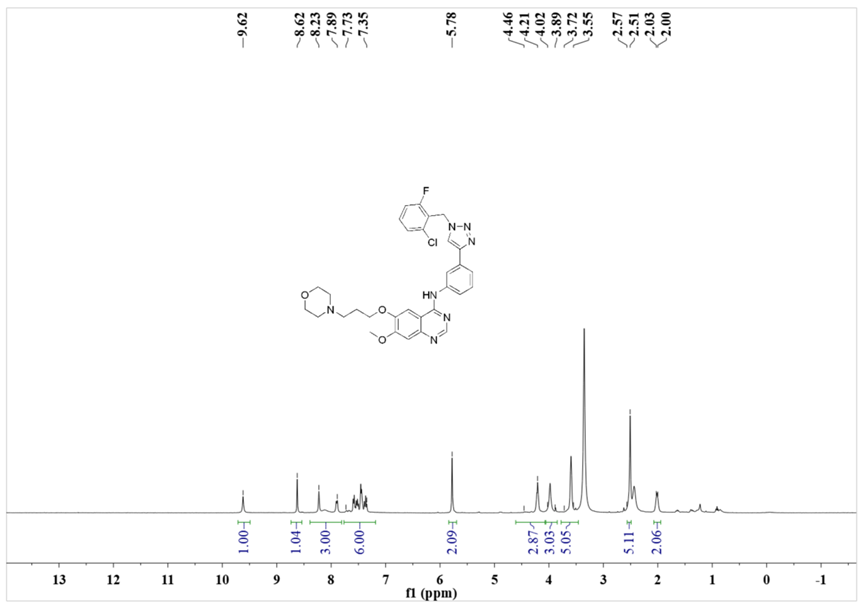


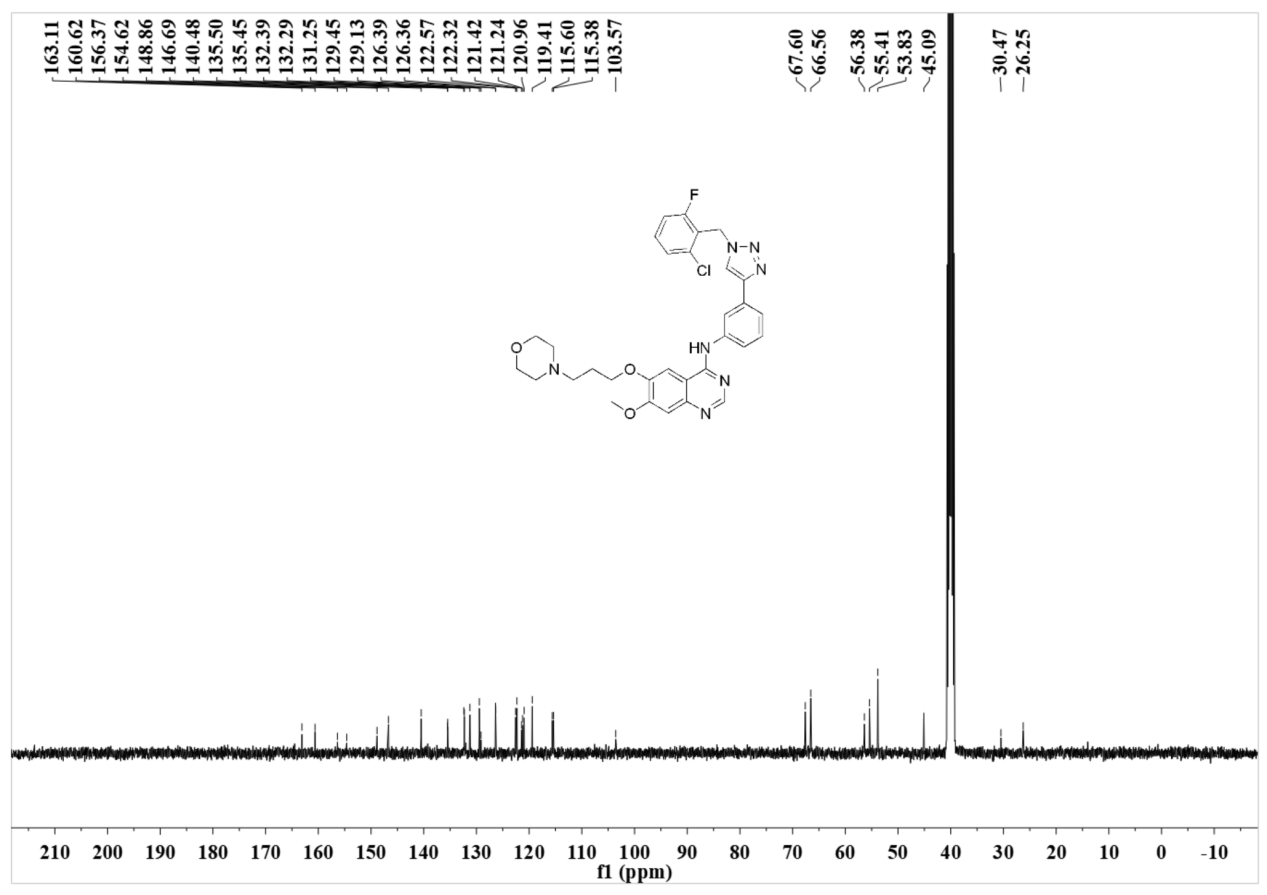


^1^H NMR and ^13^C NMR spectrums of compound **c8**


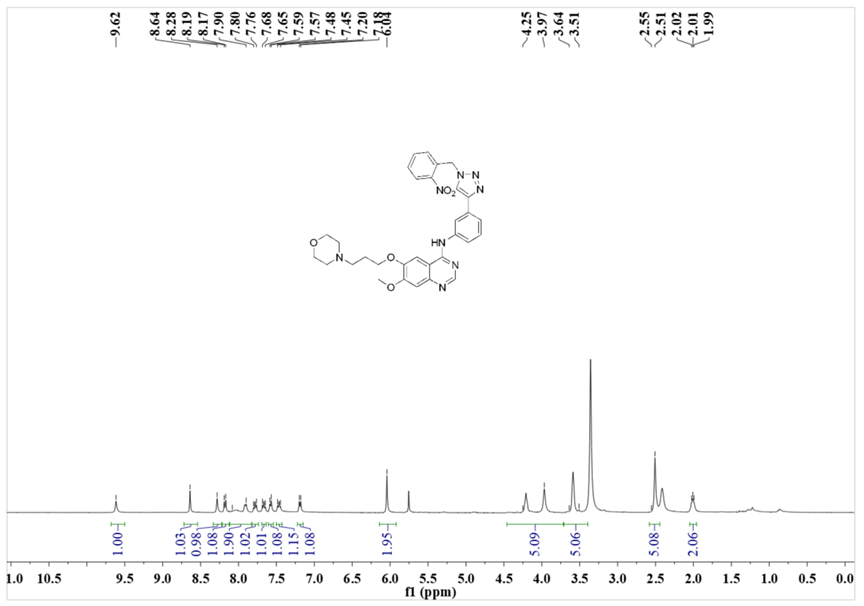


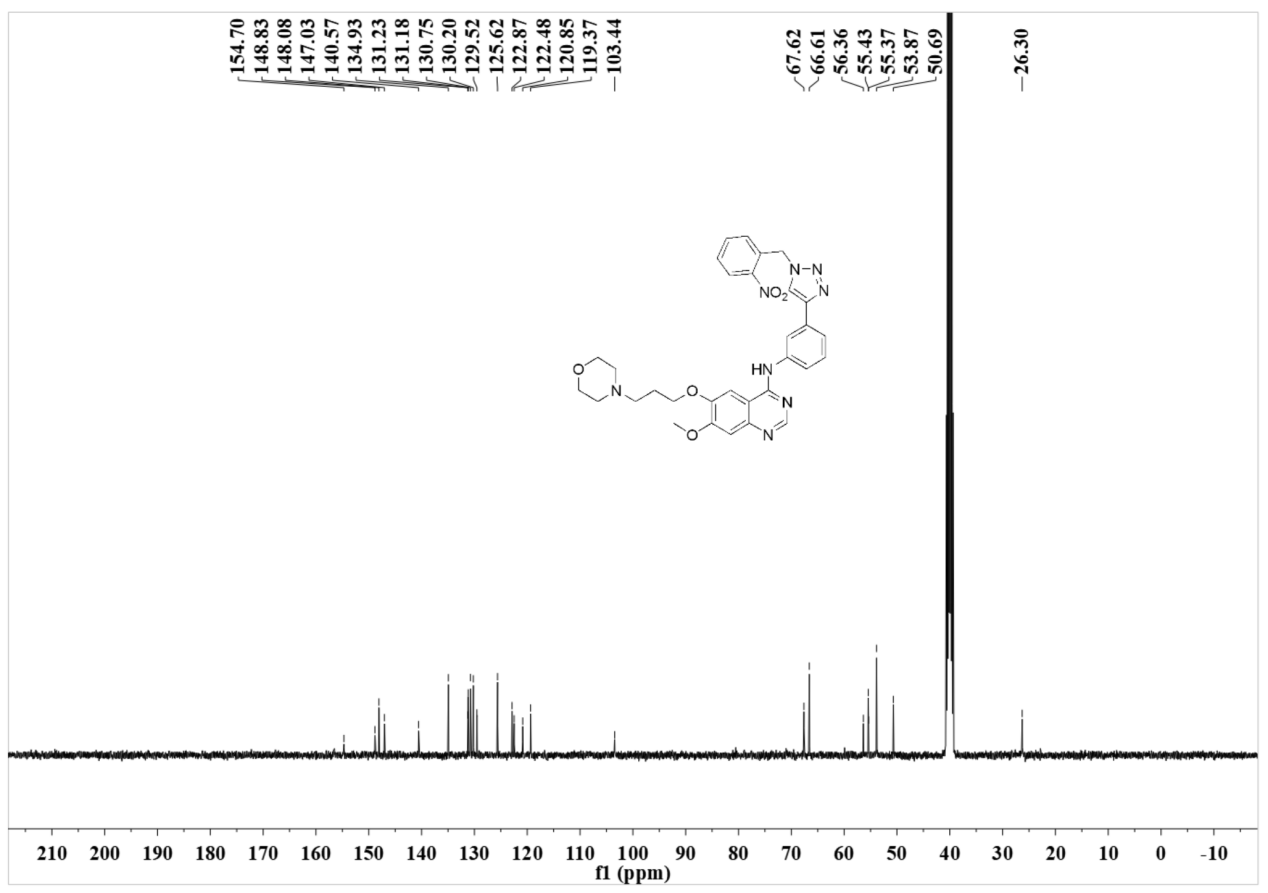


^1^H NMR and ^13^C NMR spectrums of compound **c9**


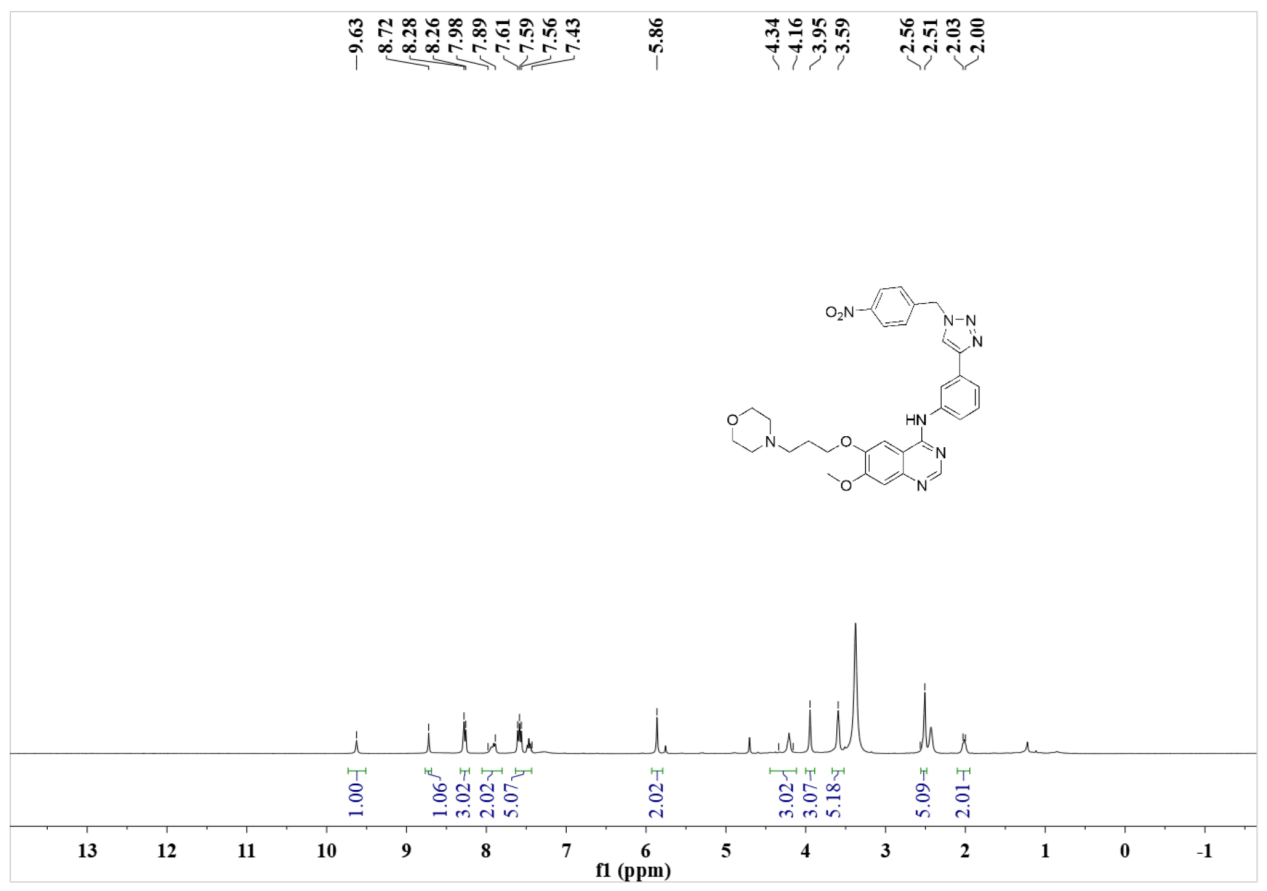


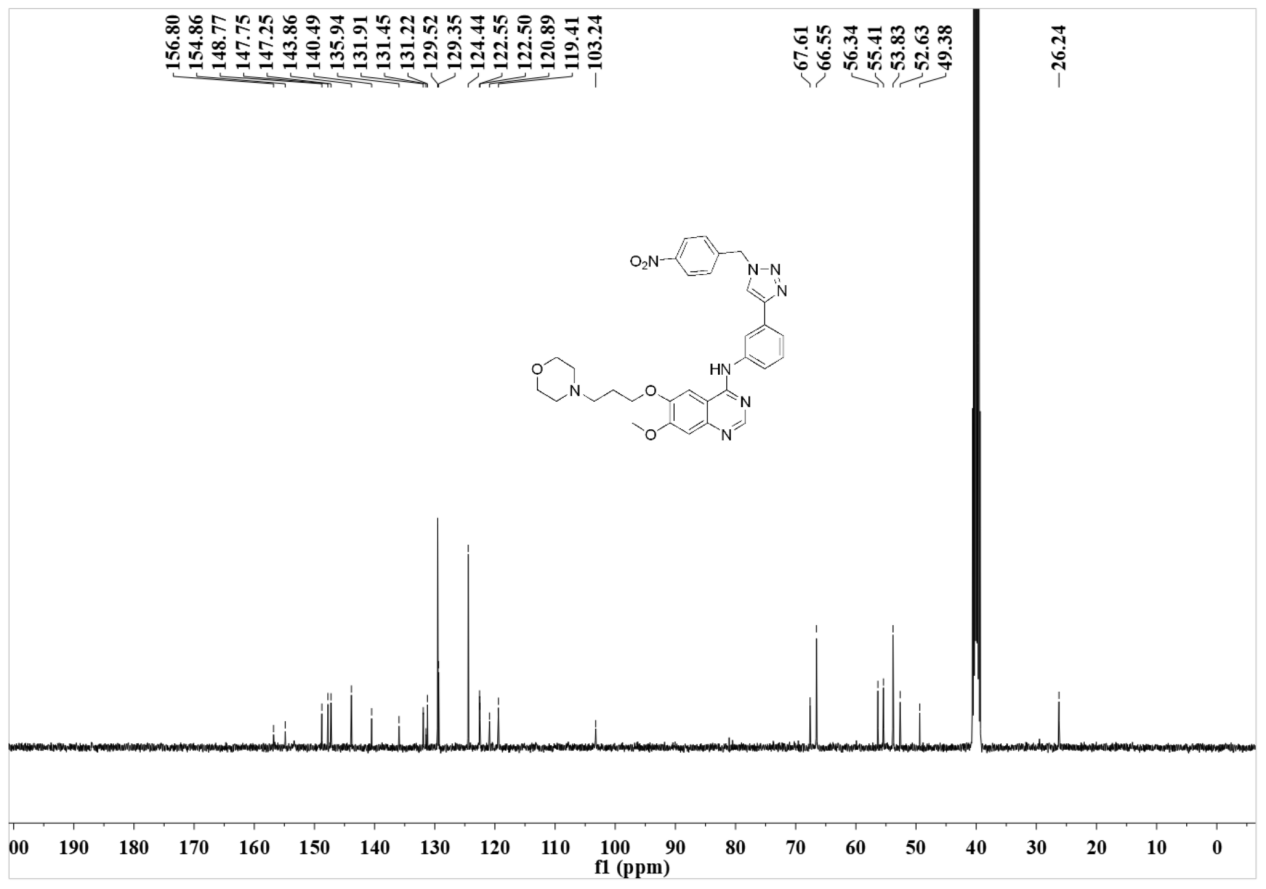


^1^H NMR and ^13^C NMR spectrums of compound **c10**


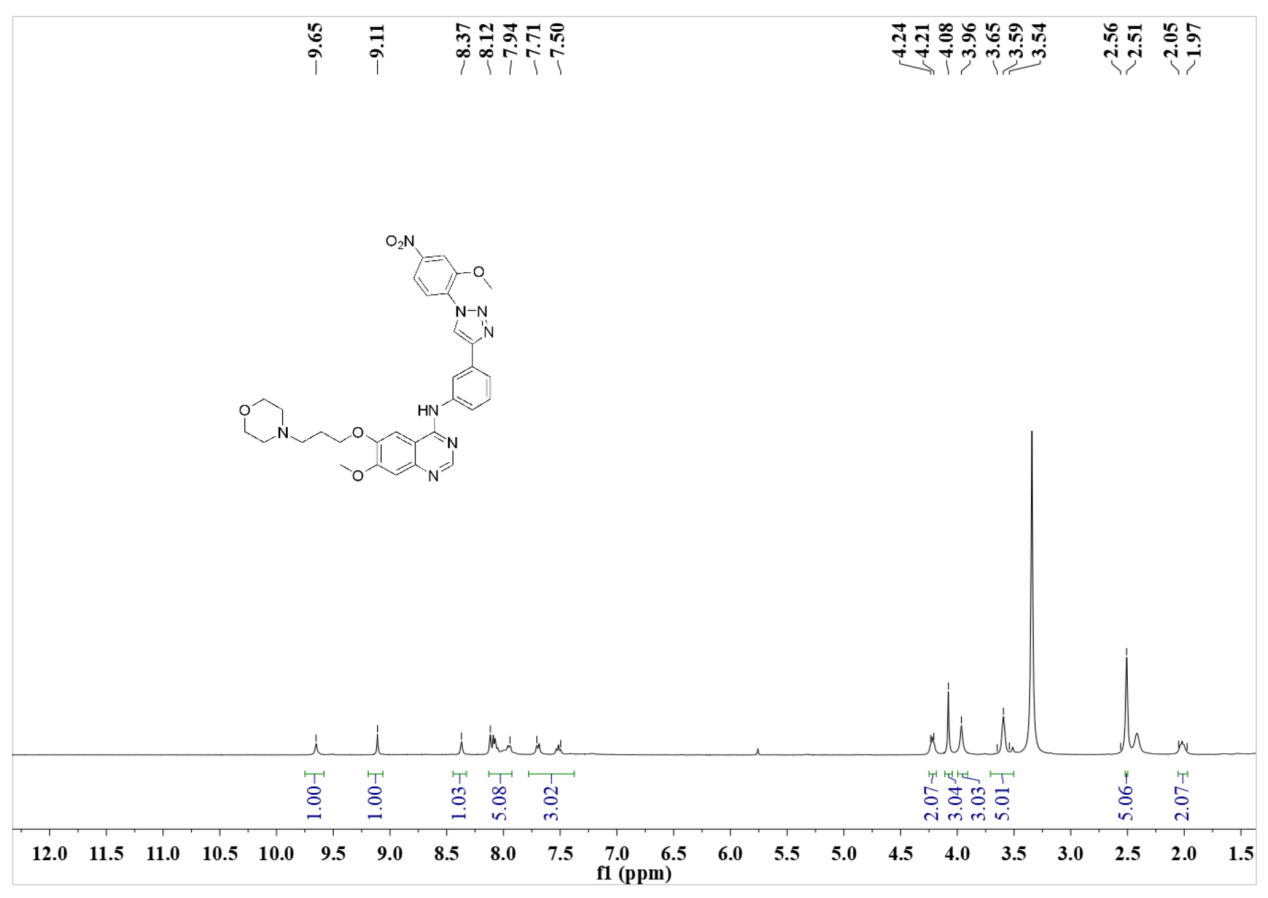


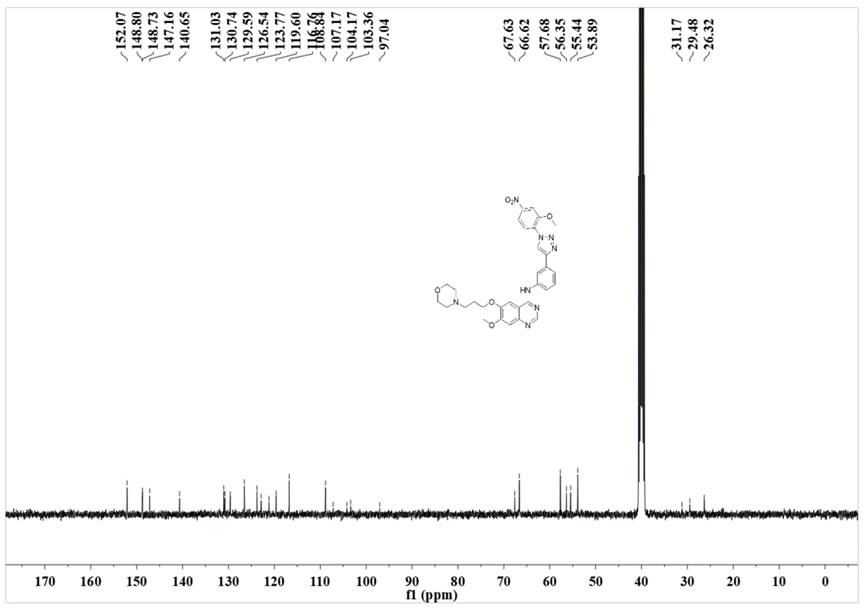


^1^H NMR and ^13^C NMR spectrums of compound **c11**

^1^H NMR and ^13^C NMR spectrums of compound **c12**


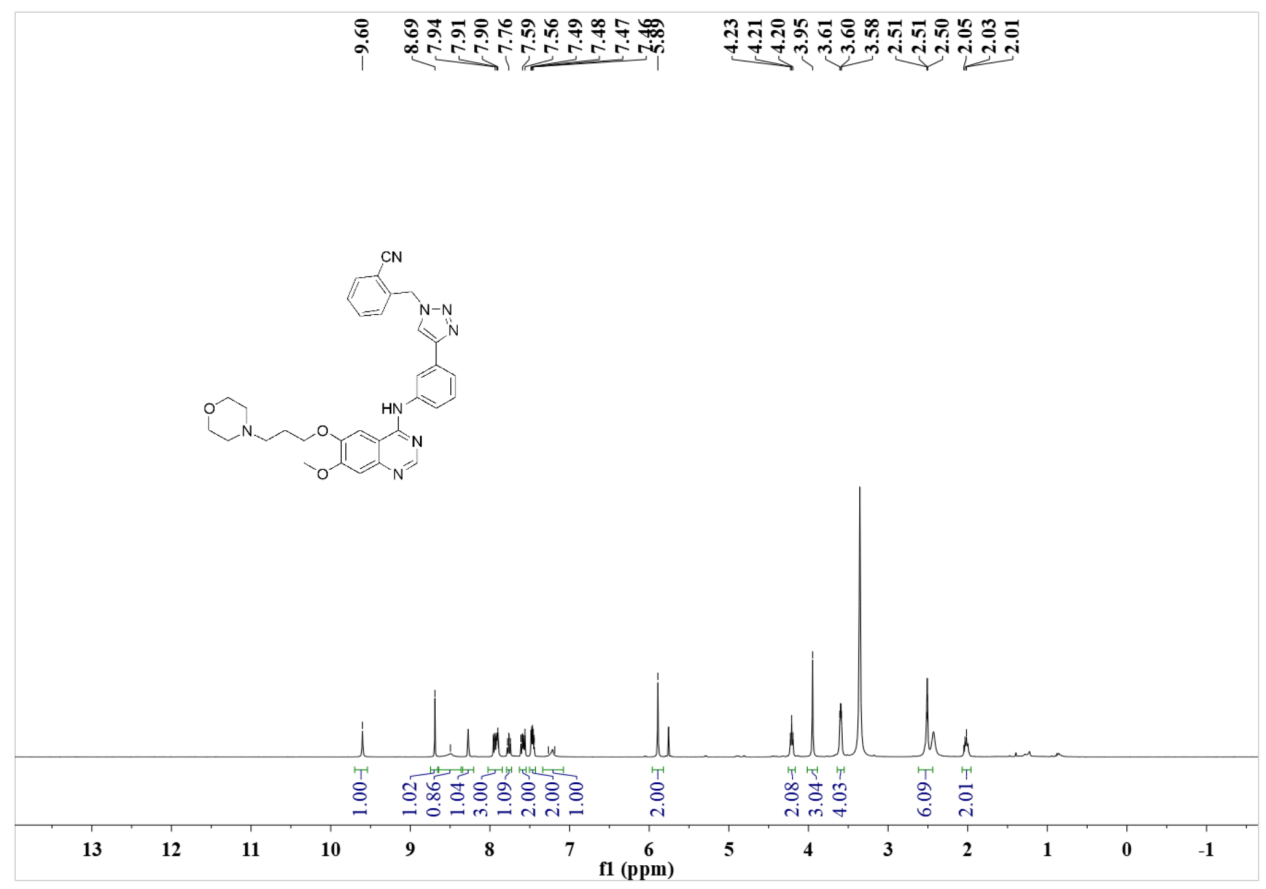


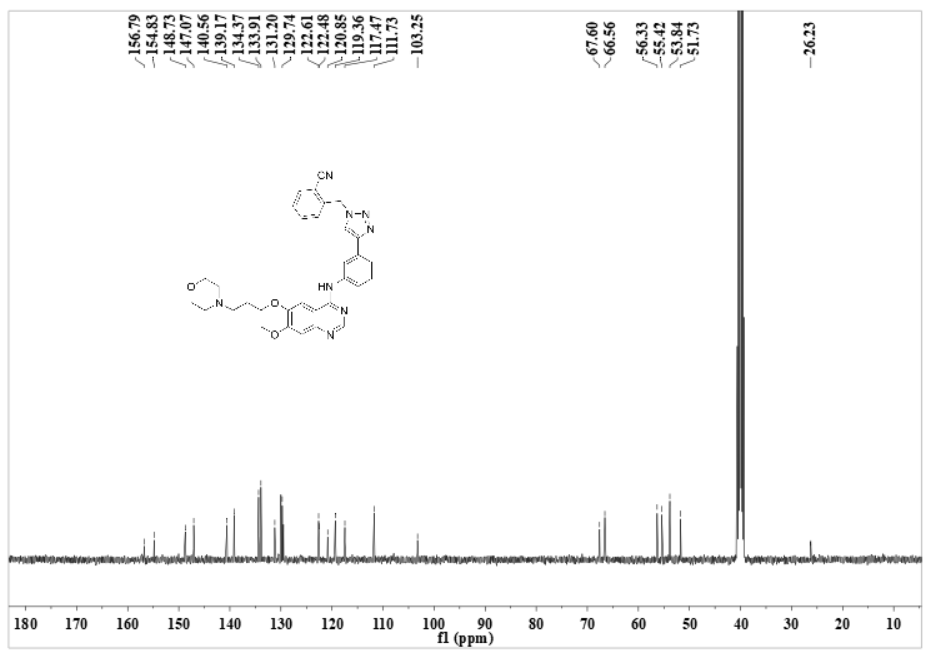


^1^H NMR and ^13^C NMR spectrums of compound **c13**


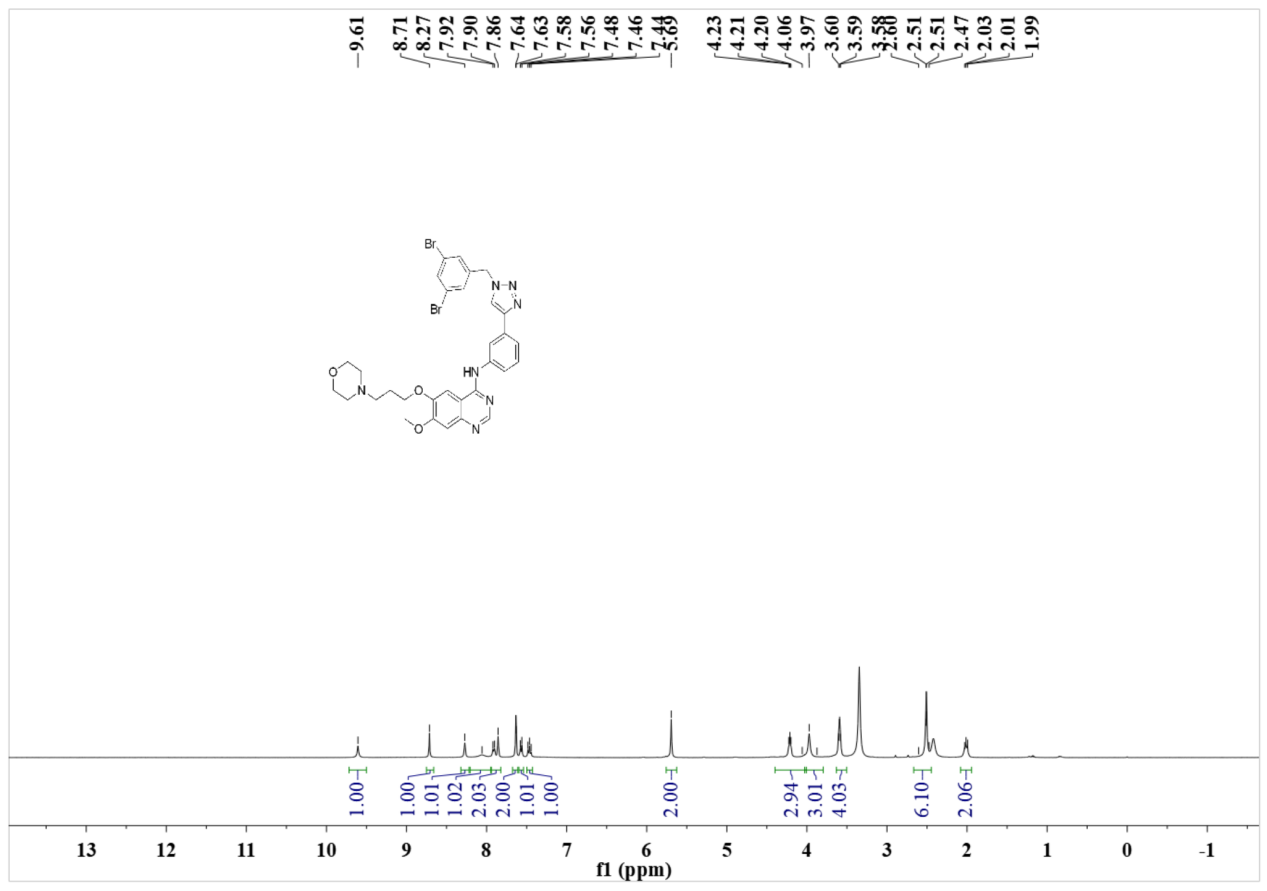


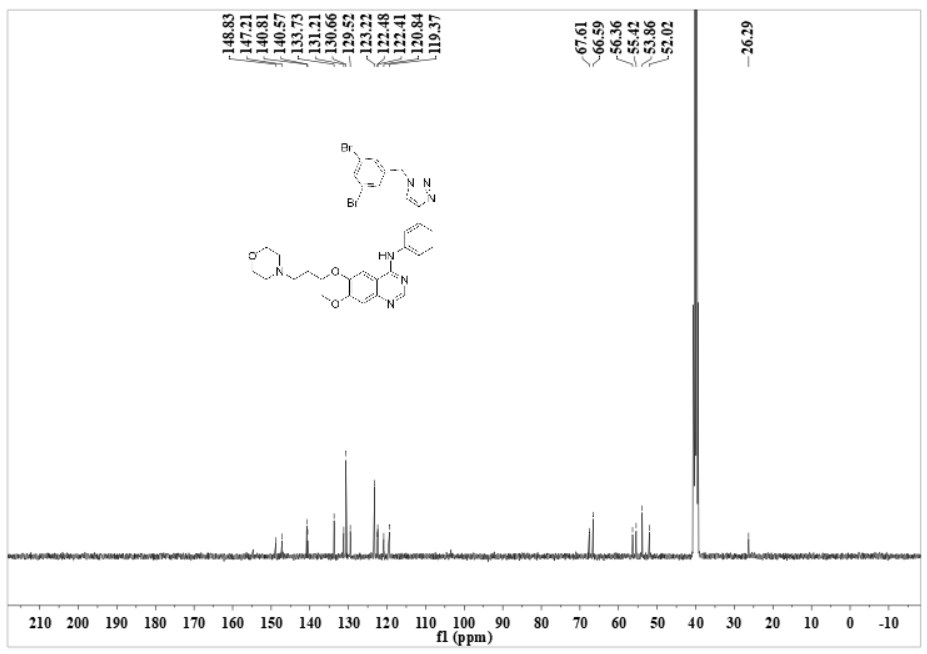


^1^H NMR and ^13^C NMR spectrums of compound **c14**


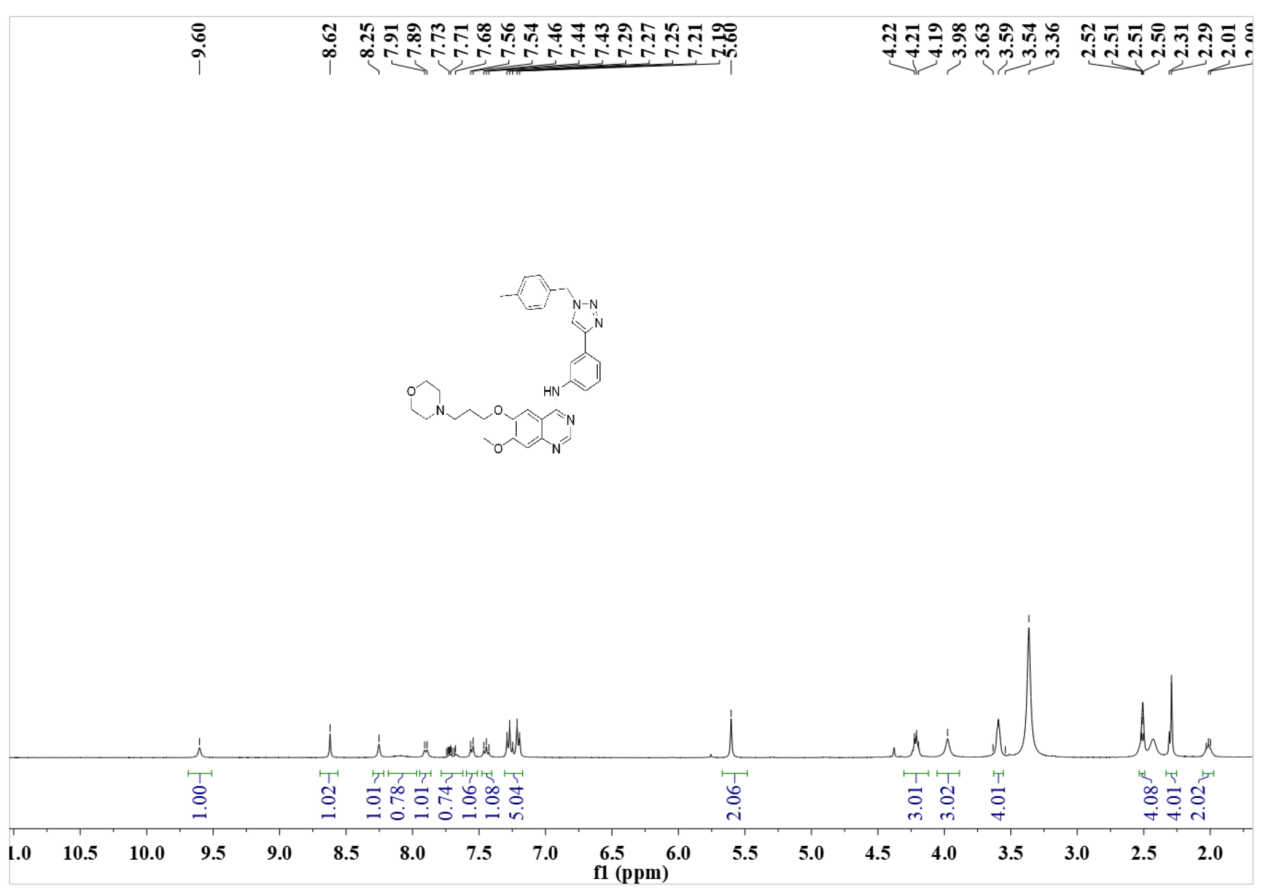


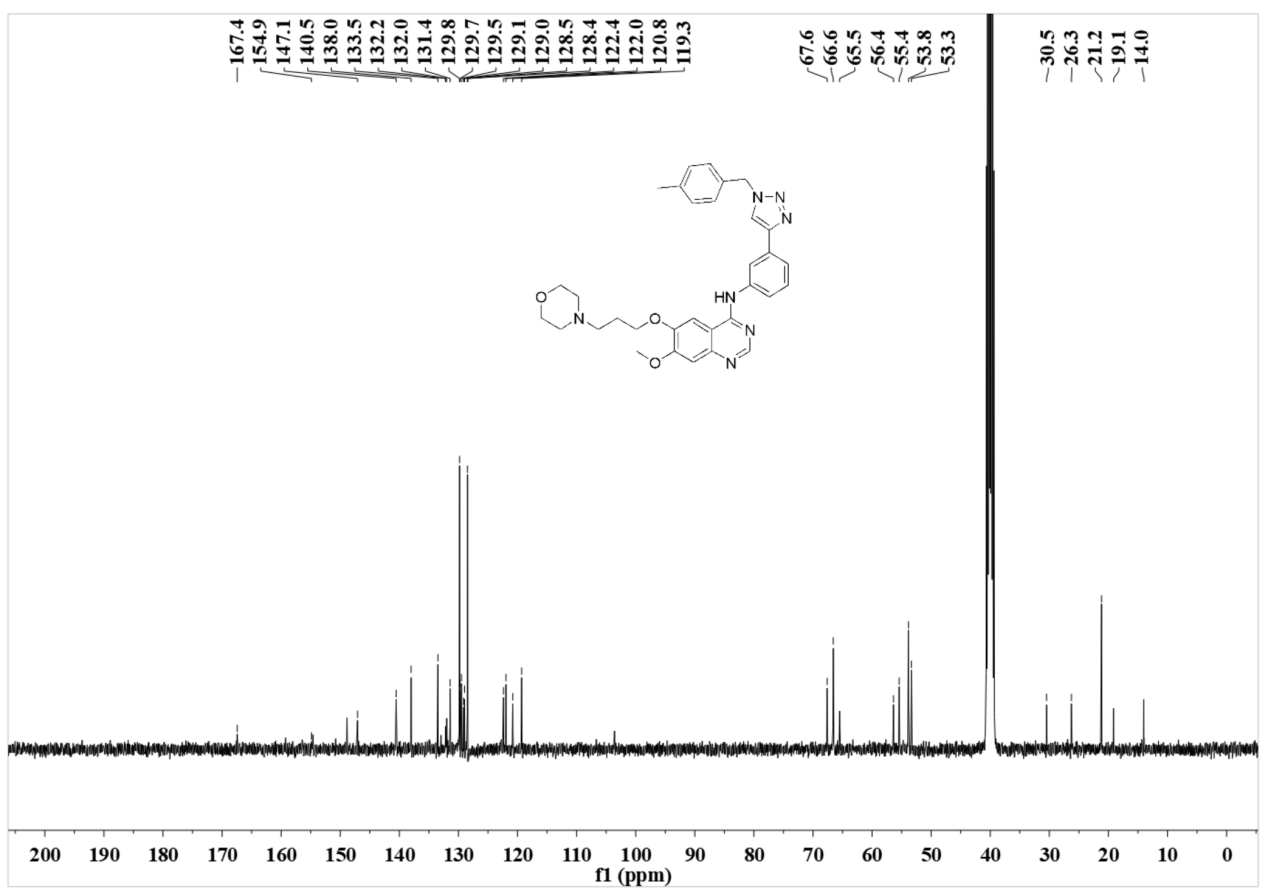

Supplement: Supplementary file 1 [file DataSheet1.docx]
